# A High Throughput In Vivo Assay for Taste Quality and Palatability

**DOI:** 10.1371/journal.pone.0072391

**Published:** 2013-08-12

**Authors:** R. Kyle Palmer, Daniel Long, Francis Brennan, Tulu Buber, Robert Bryant, F. Raymond Salemme

**Affiliations:** 1 Opertech Bio, Inc., Philadelphia, Pennsylvania, United States of America; 2 Genomind, LLC, Chalfont, Pennsylvania, United States of America; 3 Asheville Flavor Innovations, LLC, Asheville, North Carolina, United State of America; 4 Imiplex, LLC, Bristol, Pennsylvania, United State of America; Barnard College, Columbia University, United States of America

## Abstract

Taste quality and palatability are two of the most important properties measured in the evaluation of taste stimuli. Human panels can report both aspects, but are of limited experimental flexibility and throughput capacity. Relatively efficient animal models for taste evaluation have been developed, but each of them is designed to measure either taste quality or palatability as independent experimental endpoints. We present here a new apparatus and method for high throughput quantification of both taste quality and palatability using rats in an operant taste discrimination paradigm. Cohorts of four rats were trained in a modified operant chamber to sample taste stimuli by licking solutions from a 96-well plate that moved in a randomized pattern beneath the chamber floor. As a rat’s tongue entered the well it disrupted a laser beam projecting across the top of the 96-well plate, consequently producing two retractable levers that operated a pellet dispenser. The taste of sucrose was associated with food reinforcement by presses on a sucrose-designated lever, whereas the taste of water and other basic tastes were associated with the alternative lever. Each disruption of the laser was counted as a lick. Using this procedure, rats were trained to discriminate 100 mM sucrose from water, quinine, citric acid, and NaCl with 90-100% accuracy. Palatability was determined by the number of licks per trial and, due to intermediate rates of licking for water, was quantifiable along the entire spectrum of appetitiveness to aversiveness. All 96 samples were evaluated within 90 minute test sessions with no evidence of desensitization or fatigue. The technology is capable of generating multiple concentration–response functions within a single session, is suitable for in vivo primary screening of tastant libraries, and potentially can be used to evaluate stimuli for any taste system.

## Introduction

Taste is a chemosensory event that begins with the binding of exogenous chemicals to specific taste-signaling proteins in the tongue. The receptors, now well-known to be G protein-coupled receptors (GPCRs) and ion channels, and their associated signaling proteins are expressed in specialized taste cells that communicate taste signals to sensory neurons [1]. The signals that reach the brain provide information on the identity and concentration of substances in the oral cavity and on whether the substances should be ingested [2].

A variety of experimental paradigms have been established for studying taste phenomena. At the most reductionistic level of investigation are assays that rely on recombinant cell lines expressing cloned taste receptors [3]. Cell-based assays have been useful for pharmacologic characterization of the interactions between tastants and their cognate receptors and also have been used for high throughput screening of chemical libraries for discovery of novel tastants and taste modifiers [4,5]. However, measurement of emergent perceptual properties of taste, such as sensory quality and palatability, only can be obtained from the study of sentient organisms.

Studies performed with human subjects offer the advantage of indicating taste quality, intensity, and palatability by means of verbal reports, and human subjects easily can be trained to use rating scales to quantify each of these properties [6]. But conducting experiments with human taste panels can be resource-intensive and relatively limited in flexibility of experimental design. Some of these shortcomings have been circumvented by the development of reliable animal models to study complex emergent taste functions [1,7]. These models fall into two general categories: taste discrimination experiments that quantify taste quality [8–11], and studies of “taste-guided” behavior which provide measures of palatability [12–15].

Taste quality is operationally defined in taste discrimination paradigms as a measure of the degree to which the taste of a novel solution can be distinguished from that of a standard taste cue. One commonly used procedure is conditioned taste aversion (CTA), in which a standard taste cue becomes associated with an aversive stimulus, such as peritoneal injections of LiCl [16,17]. Subsequent to the conditioning, novel tastants then are avoided as a function of their similarity to the standard. In another paradigm, operant taste discrimination, subjects are trained to perform one designated behavioral task after sampling a specific standard taste cue, and an alternative task after tasting a sample that is distinguishable from the standard. For example, rodents have been trained to use food- or water-reinforced lever presses [18–20] and water-reinforced spout-licking [8,10,11] to indicate the degree of similarity or disparity between novel tastants and standard taste cues.

The property of palatability is most effectively quantified in studies of taste-guided behavior that record the rate at which an animal licks a tastant sample, usually administered through a sipper tube. The lick rates elicited by tastants are compared to those for water, yielding a measure of relative appetitiveness [21] or aversiveness [15]. Since animals usually must be fluid-deprived to motivate them to sample from the tubes, high basal lick rates for water often greatly reduce the window available for measuring tastant appetitiveness [22].

Each of these models, both by necessity or convenience, focuses either on taste quality or on palatability and studies them independently. No method previously has existed that simultaneously quantifies both taste quality and palatability, which are two separate aspects of taste. In nearly all cases of in vivo taste testing, the numbers of samples that can be evaluated in any given experiment are relatively few, particularly in comparison to those in recombinant cell-based assays.

We have invented an apparatus and methodology for high throughput taste assessment using rats, which simultaneously quantifies both taste quality and palatability. Rats are trained to sample taste stimuli presented in a standard 96-well plate and subsequently perform an operant discrimination by pressing levers for food pellets. Food-reinforced lever presses in the taste discrimination component provide the measure of taste quality, and licks from the 96-well plate are recorded to indicate palatability, on each trial. The ability to test 96 samples within a single session enables rapid generation of high quality concentration–response functions for multiple compounds, yielding accurate values for potency and efficacy. Our method also is suitable for direct in vivo primary screening of tastant libraries dispensed in 96-well plates.

## Methods

### Ethics Statement

All procedures for these experiments were approved by the Albert Einstein Healthcare Network Institutional Animal Care and Use Committee.

### Materials

Acesulfame potassium (Ace-K), trehalose, maltose, L-glycine, glycyrrhizic acid, sodium cyclamate, glucose, fructose, sucrose, alloxan tetrahydrate, zinc sulfate (ZnSO_4_), aspartame, saccharin, sucralose, citric acid, quinine hydrochloride, sodium chloride (NaCl), monosodium glutamate (MSG), and amiloride were purchased from Sigma-Aldrich (St. Louis, MO). Rebaudioside A, stevioside, luo han guo, and SC45647 were acquired from Redpoint Bio Corp (Ewing, NJ). Polycose was from Ross Nutrition (Columbus, OH). All tastants were dissolved in double-deionized distilled water purchased from Invitrogen (Grand Island, NY).

### Rats

A total of 13 Sprague Dawley male rats from Taconic (Albany, NY) were used for the experiments described herein. All rats entered the studies at 12 weeks of age (approximately 200-250 g) and were housed singly with cedar chip bedding, a red-tinted plastic cylinder (15 cm l x 7.5 cm ID), and free access to water. From the beginning of the studies, rats were kept on a maintenance energy diet [23] designed to approximate a daily balance between caloric intake and energy expenditure. Rats subjected to this diet were motivated to perform operant tasks to receive single 45 mg grain-based food pellets throughout training and test session (96 pellets per session). An additional 8 g of supplemental rodent chow (PicoLab Rodent Diet 20, Lab Diet, St. Louis, MO) per day were given in the home cage. Maintenance diet conditions remained in effect throughout the course of the experiments and all rats steadily gained weight.

A single cohort of 4 rats was used for experiments involving sweet taste responses, which spanned over a period of approximately 10 months. Additional, experimentally naïve rats were trained for the salt (one cohort of 3 rats) and umami (one cohort of 4 rats) taste experiments. Finally, 2 additional rats that were trained under the methods (described below) for sucrose discrimination were studied for validation of the lickometer function.

### Apparatus

**Figure 1:**
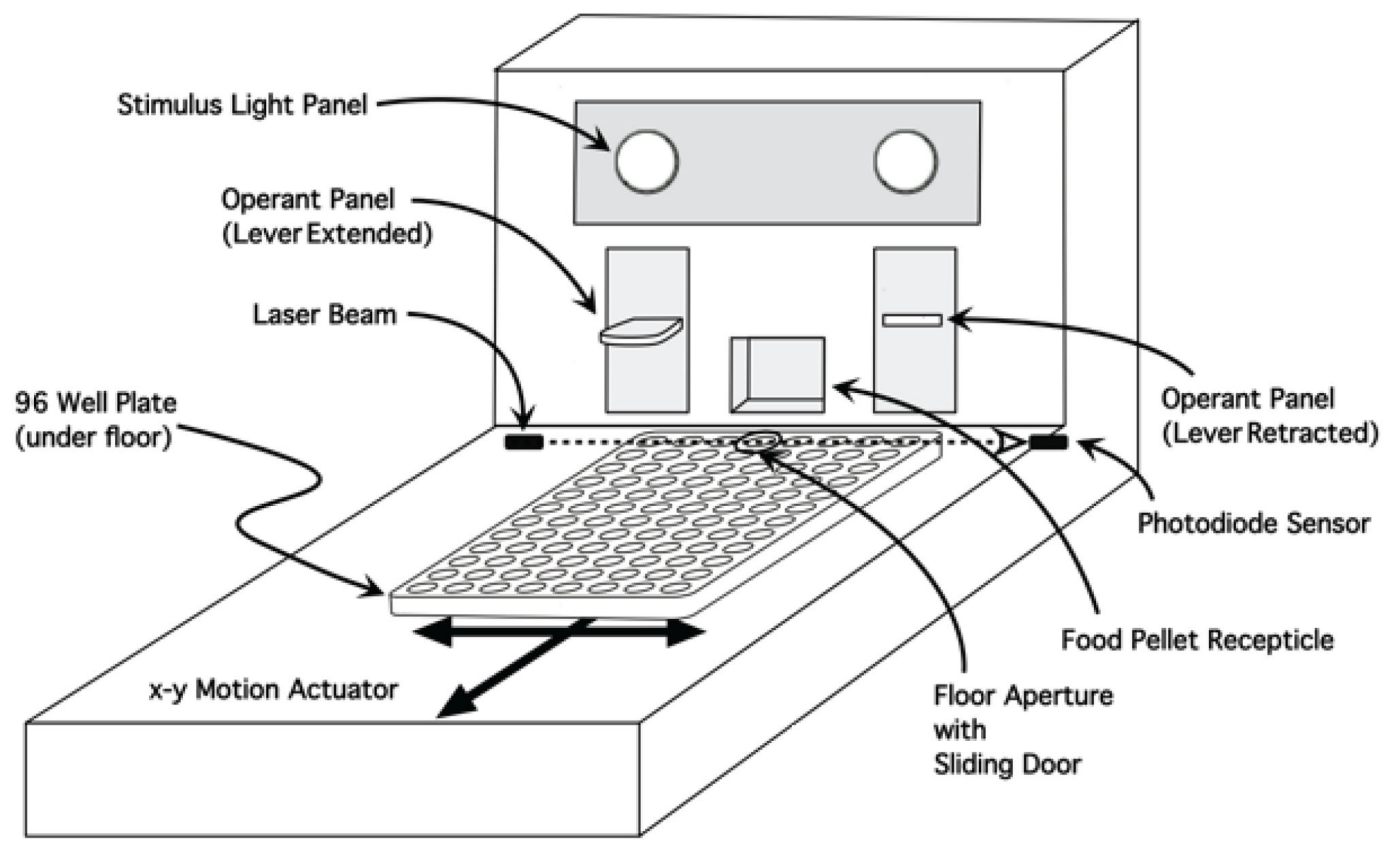
Schematic of the Apparatus. Two retractable levers in the front panel operate a food pellet dispenser. The front half of the floor swings upward to expose a sub-chamber beneath. A standard 96-well plate is placed on an x-y motion table in the sub-chamber. When the floor is closed, the contents of a single well can be accessed by licking through an aperture in the floor. Insertion of a rat’s tongue disrupts the path of a laser beam projected across the top of the well, activating a switch that produces the levers. Above each lever is a stimulus light. The chamber also contains a house-light in the ceiling and a tone generator on the rear wall (not pictured).

The apparatus (Figure 1) is a modified operant chamber with Plexiglas side walls and stainless steel front and rear walls (28 cm l x 24 cm w x 26 cm h). In the center of the base of the front wall is a receptacle for the delivery of food rewards. On either side of the central receptacle are retractable levers with a stimulus cue light above each lever. In the upper left corner of the rear wall is an audio tone generator and a house light in the center of the rear wall. A video camera is mounted in the center of the ceiling.

The rear half of the chamber floor is a grid to allow droppings and urine to fall into a collection pan beneath. The front half of the floor is a stainless steel panel attached by a hinge to the forward-most bar of the grid floor of the chamber so that it can swing upwards along an arc path to expose a sub-chamber (5 cm depth). In the sub-chamber is an x-y motion table onto which a standard 96-well plate can be snapped into place. When the steel panel floor is lowered to enclose the sub-chamber, the top surface of a 96-well plate resting on the x-y table is 2 mm beneath the closed plate floor. A laser is mounted in the right wall (from the perspective of facing the front panel) of the sub-chamber so that a beam can be projected in the 2 mm space across to the opposite side where it strikes a photocell. The motor that drives the actuator is located behind the front panel. An aperture with a diameter of 5 mm, slightly smaller than that of a single well of the 96-well plate (7.15 mm) is located 10 mm from the front edge of the floor immediately before the food receptacle. The aperture is covered by a retractable trap door that automatically slides open at the beginning of a trial to give access to the contents of the well beneath. The entire operant chamber is enclosed within a polyvinyl chloride sound attenuating chamber (75 cm l x 45 cm w x 50 cm h). Vents are in the front and rear wall, with a muffin fan placed within the front vent and a sponge filter in the rear vent, to control airflow and provide masking white noise. The interface between an external computer and all of the electrical components of the apparatus is bolted to the back of the sound attenuating chamber.

The apparatus was constructed according to our design by Advent Design Corporation (Bristol, PA).

### Procedure

#### Lever Training

Rats were shaped by successive approximation to press a single lever on a fixed ratio (FR) schedule of reinforcement. Initially, a single lever press (FR1) resulted in delivery of a 45 mg grain-based pellet reinforcer. The FR was increased gradually to a final FR10. All rats acquired the FR10 response within 3 days of lever training.

#### Well-Lever Training

Rats were trained a behavioral sequence of licking the contents of the 96-well plate through the aperture followed by lever-pressing for food. Initially, a 96-well plate containing peanut butter in all wells was placed on the x-y motion table beneath the floor and the aperture in the floor remained open. A rat was placed in the chamber and allowed to explore, which eventually led to the discovery of the peanut butter and spontaneous licking from the well. Once the behavior of licking through the aperture for the contents of the well beneath was established, the peanut butter plate was replaced with one containing 300 mM sucrose in all 96 wells. At this point, training began for chaining the well-licking behavior to lever-pressing.

Just prior to the start of the training session, the house light was off, both levers were withdrawn behind the front panel, and the aperture over the 96-well plate was covered by the sliding trap door. The x-y motion table initialized by moving the plate so that a single randomly assigned well of the 96-well plate aligned concentric with the aperture.

At the start of the session, the house light switched on to illuminate the interior of the chamber. For all training and test sessions, the beginning of a trial was marked by the simultaneous sounding of a 5 second 4.5 kHz tone and retraction of the door covering the aperture, providing access to the contents of a single well in the 96-well plate.

As the rat’s tongue entered the well, the laser beam was interrupted, actuating a switch that caused one lever to extend from the front panel and the stimulus light above the lever to be illuminated. The well remained open and in place so that rats could lick repeatedly. There was no time restriction on the trial, so that rats licked feely until they abandoned the well to perform the lever-press operant. Fully trained rats moved immediately to lever pressing upon completion of licking.

The aperture door closed over the well upon the first lever press and the x-y motion table moved the plate to align the next well in the random sequence concentrically with the aperture. Completion of the FR lever press requirement on the appropriate lever resulted in delivery of 45 mg food pellet reinforcer into the receptacle ending the trial. There was no consequence for failing to complete the FR; that is, there was no limited hold on responding. Thus trial duration was under the control of the rat, which was motivated to complete the behavioral chain as quickly as possible to obtain a food pellet. Upon completion of a trial the lever was withdrawn, the stimulus light extinguished, and a 30 second inter-trial interval began. The well-lever behavioral sequence was established in all rats within 5 additional days of training.

#### Discrimination Training

Half of the wells of a 96-well plate were filled with 100 mM sucrose and the other half with water, and then the plate was placed on the x-y motion table. The trial sequence described above under *Well-Lever* training was identical except that two levers were produced upon licking from the well. One lever was designated the “sucrose lever” and the other as the “non-sucrose” lever. On any given trial, if the well contained sucrose the stimulus light above the sucrose-lever was illuminated and only presses on the sucrose-lever resulted in food delivery. The light above the non-sucrose lever remained off and presses on the non-sucrose lever were not reinforced. The converse was true for water trials. A 30 second inter-trial interval began if the FR10 was completed on the appropriate lever (resulting in reinforcement.) Completion of an FR10 on the incorrect lever did not produce a food pellet but instead resulted in a 60 second timeout, during which the house light was extinguished. Again, as in the *Well-Lever Training* procedure, there was no limited hold on responding.

When lever-appropriate responding reached 90%, training continued with stimulus lights illuminated above both levers in all subsequent sessions. New taste stimuli, beginning with 100 mM NaCl, were added to the discrimination training regimen once 90% accurate discrimination between sucrose and water was established. Beginning with NaCl and followed sequentially by 10 mM citric acid then 1 mM quinine on subsequent sessions, all additional tastants were added as stimuli with contingency for reinforcement on the non-sucrose lever. When a new tastant was added to the training regimen, lever-appropriate responding was allowed to return to 90% within a session before adding the next tastant in a subsequent session. Each well was presented once for a total of 96 trials per session. By this stage, failure to complete a trial during a session marked mechanical malfunctions. Thus the rats controlled the duration of the sessions, which generally required approximately 90 minutes or less for complete evaluation of all 96 wells.

Rats were considered ready for testing novel tastants when at least 90% of responses on sucrose trials were made on the sucrose-lever and 90% of responses on NaCl, citric acid, quinine, or water trials occurred on the non-sucrose lever for at least two consecutive training sessions. The entire process of training the rats to test-readiness in the discrimination task, including the training required for establishing the well-licking to lever-pressing behavioral sequence, required between 4–7 weeks (depending on the rat.)

#### Testing

The procedure for testing the properties of novel tastants was identical to that described above under *Discrimination Training*, except that the contingency for reinforcement on responses appropriate to sucrose- and non-sucrose levers was in place only on control trials. Thus on control trials, an incorrect choice was not reinforced and resulted in a timeout, and on trials in which test articles were presented, responding on either lever resulted in delivery of a food pellet.

#### Plate Configurations for Testing

Numerous configurations of test articles and controls are possible with the matrix of a 96-well plate format. We have found that a configuration consisting of as few as 4 wells devoted to each of NaCl, quinine, and citric acid, and 6 wells to each of water and sucrose (i.e., a total of 24 controls which required a correct response for reinforcement) leaving available 72 wells for test article evaluation (in which presses on either lever were reinforced) was sufficient to maintain 90-100% control accuracy throughout test sessions (note: this particular configuration was not used for the experiments described herein.) For any given experiment described herein, test articles were dispensed in as many wells as were required to evaluate the tastant of interest and all remaining wells were distributed as evenly as possible among the control taste stimuli. If the distribution of test articles resulted in a remainder of wells that could not be evenly divided among the controls, then NaCl, quinine, and citric acid were equally distributed and the extra wells were divided between sucrose and water. Plate configurations for all of the taste discrimination experiments are given as supplemental information in Figures S1-S10.

#### Salt and Umami Taste Discriminations

The procedures for establishing both assays followed the protocol established for sucrose discrimination, except that appropriate taste stimuli replaced 100 mM sucrose as the discriminatory cue, and sucrose in turn was trained as a negative control. For the salt taste test, 100 mM NaCl was moved from the group of negative controls to become the discriminatory stimulus for training. For the umami taste test, the discriminatory cue was 100 mM MSG + 100 µM amiloride (added to minimize the salt taste imparted by MSG).

### Data Analysis

Taste quality was determined by the percentage of presses that occurred on the lever designated for the training taste cue on each trial. Palatability was determined by the number of licks per trial. All data were averaged across the 3-4 rats within a cohort. Student’s t test was used to determine significant differences between two means, and one-way analysis of variance (ANOVA) for evaluating significant effects among multiple means (GraphPad Prism, La Jolla, CA); when ANOVA indicated significance, comparisons between pairs of means were evaluated by Tukey’s Multiple Comparison Test. Curve fitting to datasets was achieved by linear or nonlinear regression (GraphPad Prism) where appropriate. For concentration–response functions, EC_50_ values and 95% confidence intervals (CI95%) were derived from the curve fit. Statistical determination of differences between pairs of concentration–response functions was achieved by extra sum-of-squares F test, with the log EC50 and Hill slope selected as the parameters used as the basis for the comparisons (GraphPad Prism). Values reported in the text are mean with standard error of the mean (SEM).

## Results

### Acquisition of Taste Discrimination

A cohort of 4 rats first was trained to discriminate the taste of a 100 mM sucrose solution from water. On the first days of discrimination training, responses were evenly distributed across the two levers indicating that no association between the sucrose taste and reinforcement of responses on the sucrose-appropriate lever was in effect. Although only a single lick for any trial was required to gain access to the levers that operated the food hopper, rats continued to lick. Lick rates were significantly higher for 100 mM sucrose than for water on the first exposure (mean sucrose licks/trial = 33, SEM = 3; mean water licks/trial = 9, SEM = 2, p <0.0001, Student’s t). All rats completed the 96 trials in less than 90 minutes with no evidence of desensitization or fatigue.

**Figure 2:**
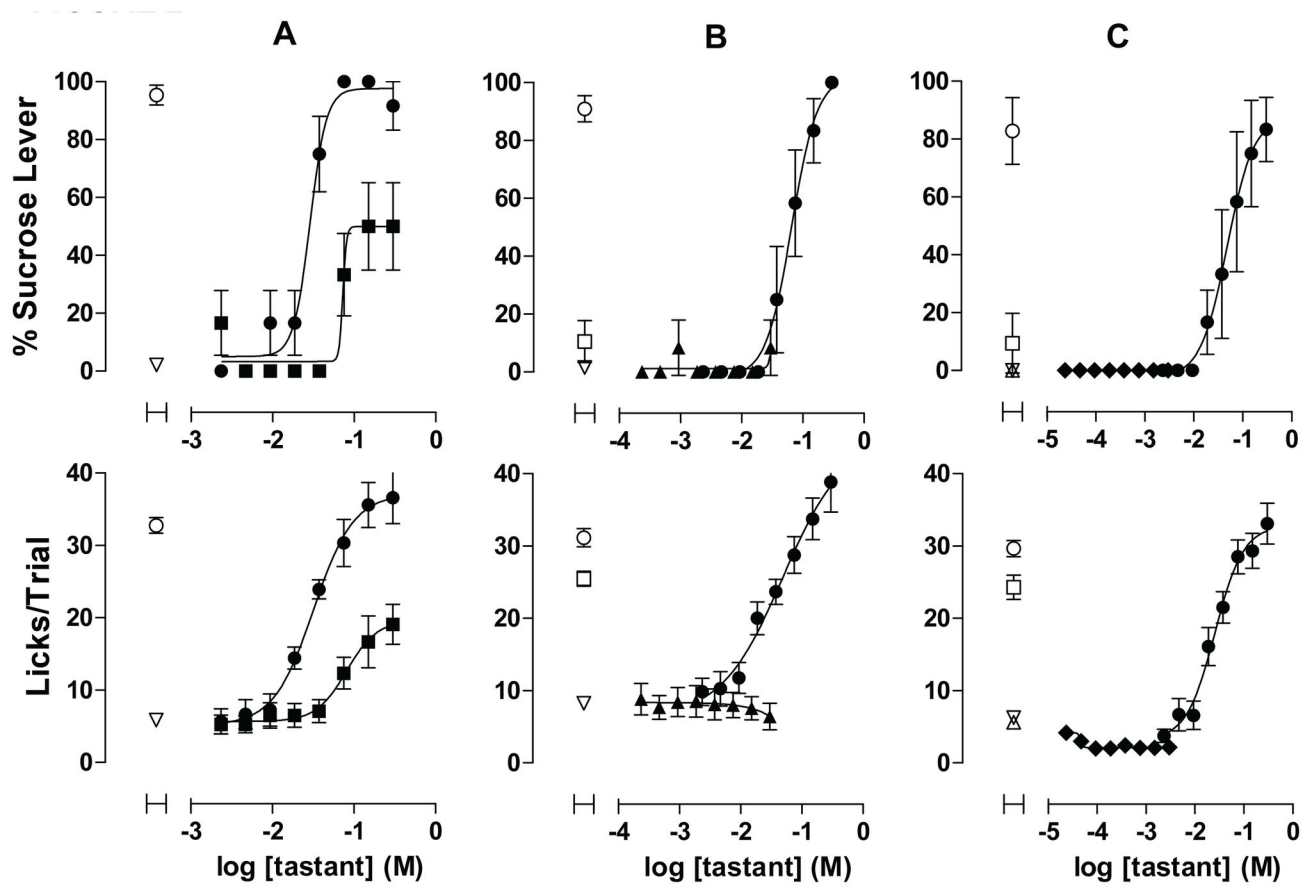
Acquisition of Taste Discrimination. Rats were trained to discriminate sucrose from water and then were challenged with additional non-sweet tastants. Upper graphs in each panel show sweet taste quality plotted as percent of responses made on the sucrose lever. Lower panels show concentration–response functions for palatability plotted as mean licks per trial. Data are plotted as mean of responses to contents from 3 wells for test articles and 12-24 wells per control tastant per rat, averaged across 4 rats. Error bars are SEM. Open symbols represent values obtained for control tastants: ○ = 100 mM sucrose; ▽ = water; □ = 100 mM NaCl; △ = 10 mM citric acid; ◇ = 1 mM quinine. Closed symbols = test articles: ● = sucrose; ■ = NaCl; ▲ = citric acid; ◆ = quinine. **A**: NaCl concentration–response function after rats had established discrimination between 100 mM sucrose and water. **B**: Citric acid concentration–response function after discrimination of sucrose from water and salt tastes established. **C**: Quinine concentration–response function after discrimination of sucrose from water, salt, and sour tastes was complete. Results in each of panel are representative of two identical experimental designs performed with the same 4 rats. See Figure S1-S3 for plate configurations corresponding to the experiments illustrated in Figure 2A–2C.

With repeated exposure over days of training the contingency between the discriminative stimulus and reinforcement of sucrose-appropriate responding, differential distribution of lever presses gradually came under stimulus control (Figure 2A, upper panel). By day 15 of discrimination training, all rats in the cohort had achieved 90% or greater sucrose-appropriate responding (mean = 12 days, SEM = 2.)

Varying the concentration of sucrose by successive two-fold dilutions, starting with a high concentration of 300 mM, resulted in a concentration–response function for both the sensory discrimination and the lick rate. Since the discrimination that had been established through training up to this point had been between 100 mM sucrose and water, the rats’ lever-pressing behavior could have been directed by stimulus intensity and not sweet taste quality. Therefore we introduced a concentration range of NaCl solutions as a novel taste to test the possibility that taste intensity was the discriminatory cue. As shown in Figure 2A (upper panel), rats responded to the two highest concentrations of NaCl (150 and 300 mM) with 50% (SEM = 22%) of presses occurring on the sucrose lever, indicating that taste intensity contributed substantially to the discriminatory cue.

Discrimination training then resumed with food-reinforcement contingent upon responding on the non-sucrose lever when sample wells contained either water or 100 mM NaCl. Training continued for an additional 5 days, by the end of which at least 90% of the responses occurred on the sucrose lever for sucrose trials and 10% or less during water or NaCl trials. When the discrimination of sucrose from both water and 100 mM NaCl solution was established, a concentration range of citric acid was tested. As shown in Figure 2B (upper panel), nearly all of the responses to citric acid at any concentration occurred on the non-sucrose lever, indicating that rats were attending to taste quality as the predominant discriminatory cue. A slight reduction in lick rates relative to water that appeared at 15 mM and was more pronounced at 30 mM (Figure 2B, lower panel) indicated that citric acid was impacting rat taste in this concentration range (results consistent with those reported elsewhere [8]).

**Figure 3:**
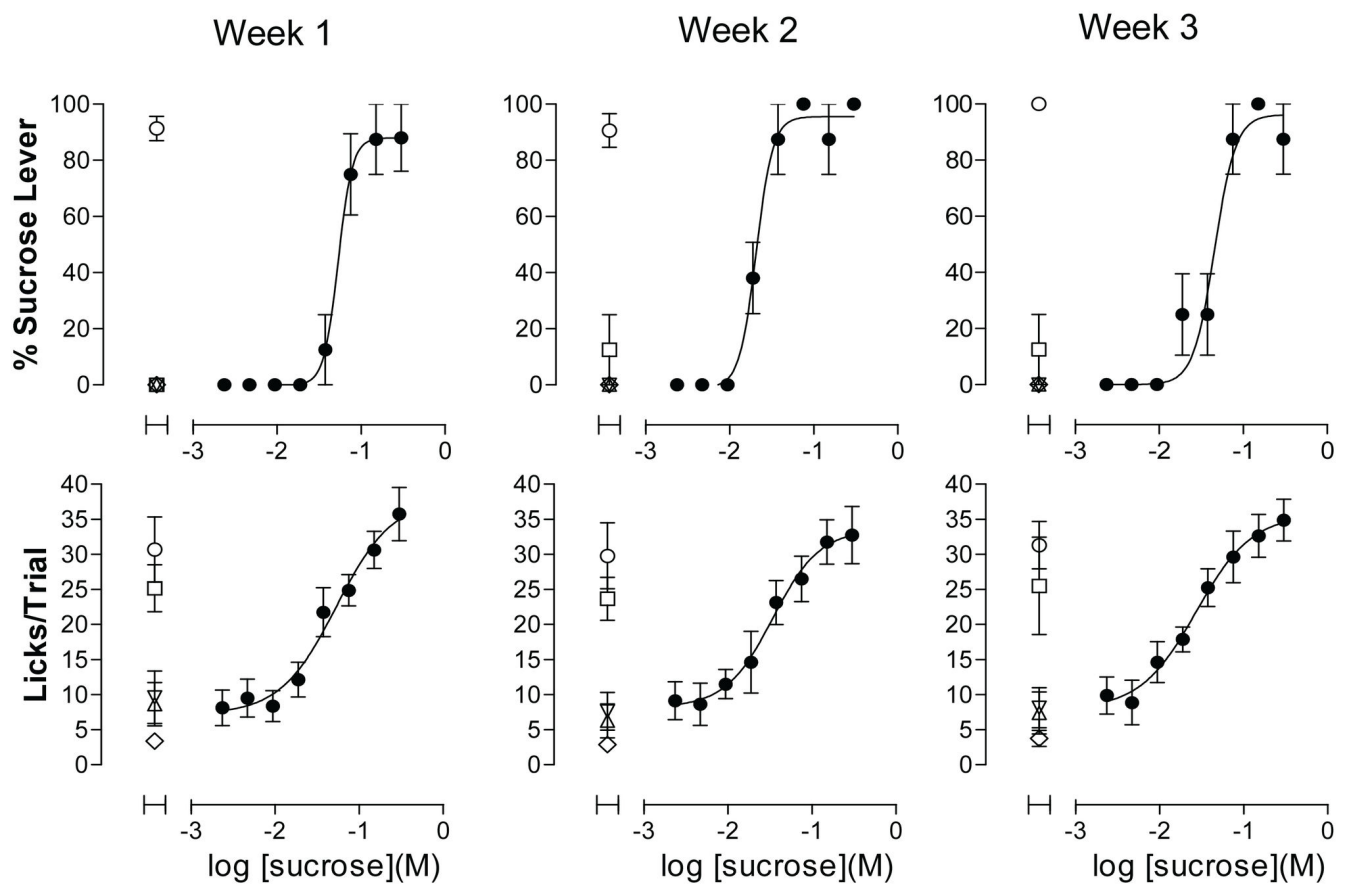
Concentration–response Functions are Stable Across Tests Following Completion of Discrimination Training. Upper graphs in each panel show sweet taste quality plotted as percent of responses made on the sucrose lever. Lower panels show concentration–response functions for palatability plotted as mean licks per trial. Data are plotted as mean of responses to contents from 4 wells for sucrose concentration range and 12-14 wells for controls per rat, averaged across 4 rats. Error bars are SEM. Open symbols represent values obtained for control tastants: ○ = 100 mM sucrose; ▽ = water; □ = 100 mM NaCl; △ = 10 mM citric acid; ◇ = 1 mM quinine. Closed symbols = test article: ● = sucrose. Results in each panel are of single experiments performed in sequential weekly intervals. See Figure S4 for plate configuration corresponding to the experiment illustrated in Figure 3.

Discrimination training then continued again with the addition of 10 mM citric acid as a non-sweet control tastant. Upon establishing water, NaCl, and citric acid as negative controls for sucrose-appropriate lever pressing, the process was completed by adding quinine under the same procedure (Figure 2C). Discrimination training was considered complete when rats consistently responded with 90% or greater sucrose-appropriate responding with 100 mM sucrose as the sweet control, and 100 mM NaCl, 10 mM citric acid, 1 mM quinine, and water as the non-sweet controls. Glutamate, considered to be the quintessential umami tastant [24], was excluded from the discrimination procedure since previous reports have indicated that some characteristics of glutamate taste overlap with sweet taste in rats [16], and because recent literature suggests that umami taste is not reducible to the activity of a single receptor, but resultant from the activity of multiple taste-signaling pathways [25–27]. The concentration–response functions for sucrose in fully trained rats was stable across tests, with little variability detected in the EC_50_ values for either taste quality or palatability from week to week (Figure 3).

### Validation of Lick Measurements

To ensure that the detected disruptions of the laser beam was due to the tongue entering the well and not caused by some other means of interference, lick measurement was validated by two methods.

Video recordings (given as supplemental information in Videos S1 and S2) of a trained rat licking from a well containing 300 mM sucrose were obtained. The first video clearly shows that the size and location of the aperture make inadvertent insertion of any anatomical feature other than the tongue highly improbable. The second video was obtained by exposing the sub-chamber to allow a view of the x-y motion table and the 2 mm space between the top of the 96-well plate and the bottom of the operant chamber floor. By zooming in the video field from this angle, a recording was obtained that clearly shows the rat’s tongue (illuminated by the laser beam) appearing through the aperture, entering the 96-well plate and withdrawing back above the floor. Slow motion examination of the video indicated that the tongue disrupts the laser path 51 times in this trial, which corresponded to the number of licks recorded by the computer. The 51 licks occurred in rapid succession over an 11 second time interval. Visual inspection of the video suggests a topographical pattern to the licking behavior that includes “bursts” of licks as has been reported elsewhere in studies using lickometers [28,29]. Fine temporal resolution of licking topography was not achievable through the software running the apparatus and recording the data.

**Figure 4:**
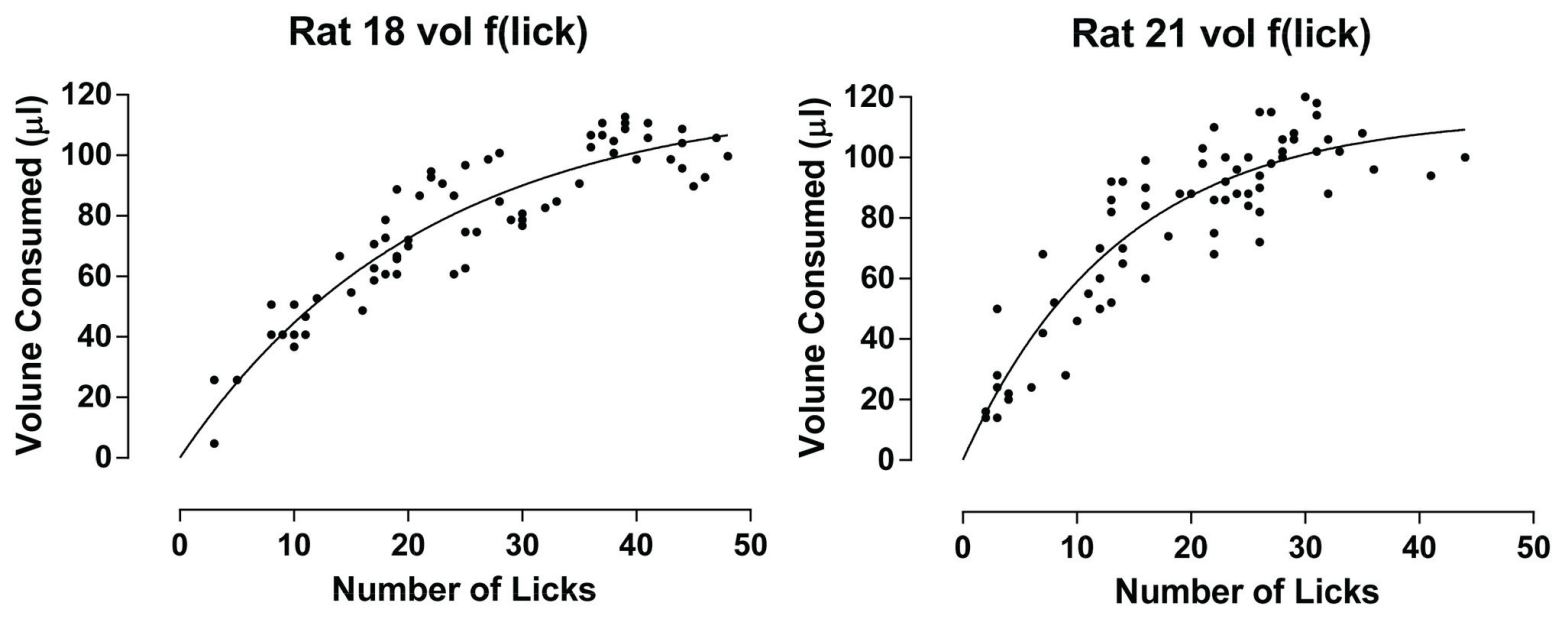
Function Relating Licks to Consumption. Licks recorded during individual trials of water and 100 mM sucrose over 3 sessions (12 each of water and sucrose per session, total of 36 water and sucrose trials) are plotted as a function of volume consumed on each trial. Shown are the data obtained from 2 rats tested on 3 consecutive days.

To further validate and characterize the lick measurements, the volume of each well was determined before and after a series of trials in order to establish a functional relationship between the number of licks and the volume of liquid consumed. Two newly trained rats (designated rat 18 and rat 21) were presented 24 randomized trials, 12 each of water and 100 mM sucrose, in 3 independent sessions. At the start of the sessions, all wells contained 290 µl of liquid. Upon completion of each session, the volume of liquid remaining in the wells was measured using a microsyringe (100 µl volume). The volume remaining in the well was subtracted from the original 290 µl to determine the volume withdrawn (presumed to have been consumed) on each trial. For each rat, the number of licks and the volume consumed on each trial was gathered from all three sessions and plotted as a function relating the two variables. The meniscus of the well contents would be expected to drop with each lick, and consequently, each successive lick should withdraw less liquid. Such a function could be quantified by a non-linear regression model if the lick rates across trials ranged sufficiently for rigorous curve fitting. The function for both rats (shown in Figure 4) was quantified using an exponential one phase association model:

$$Y=Y_{\max}(1-e^{-kX})$$

Where *Y* = lick/trial, *X* = volume consumed (μl), k = observed “rate” constant expressed as μl^-1^, and *e* = base of the natural logarithm.

Extrapolation from the functions, which were slightly different between the two rats, indicated that on the first lick of any given trial rat 18 withdrew approximately 6 µl, and rat 21 withdrew approximately 8 µl. Every subsequent lick withdrew progressively less volume. The maximum lick/trial values for each rat was attained when approximately 110 µl was consumed, probably representing the limit of depth into the well the rats’ tongues could effectively reach.

**Figure 5:**
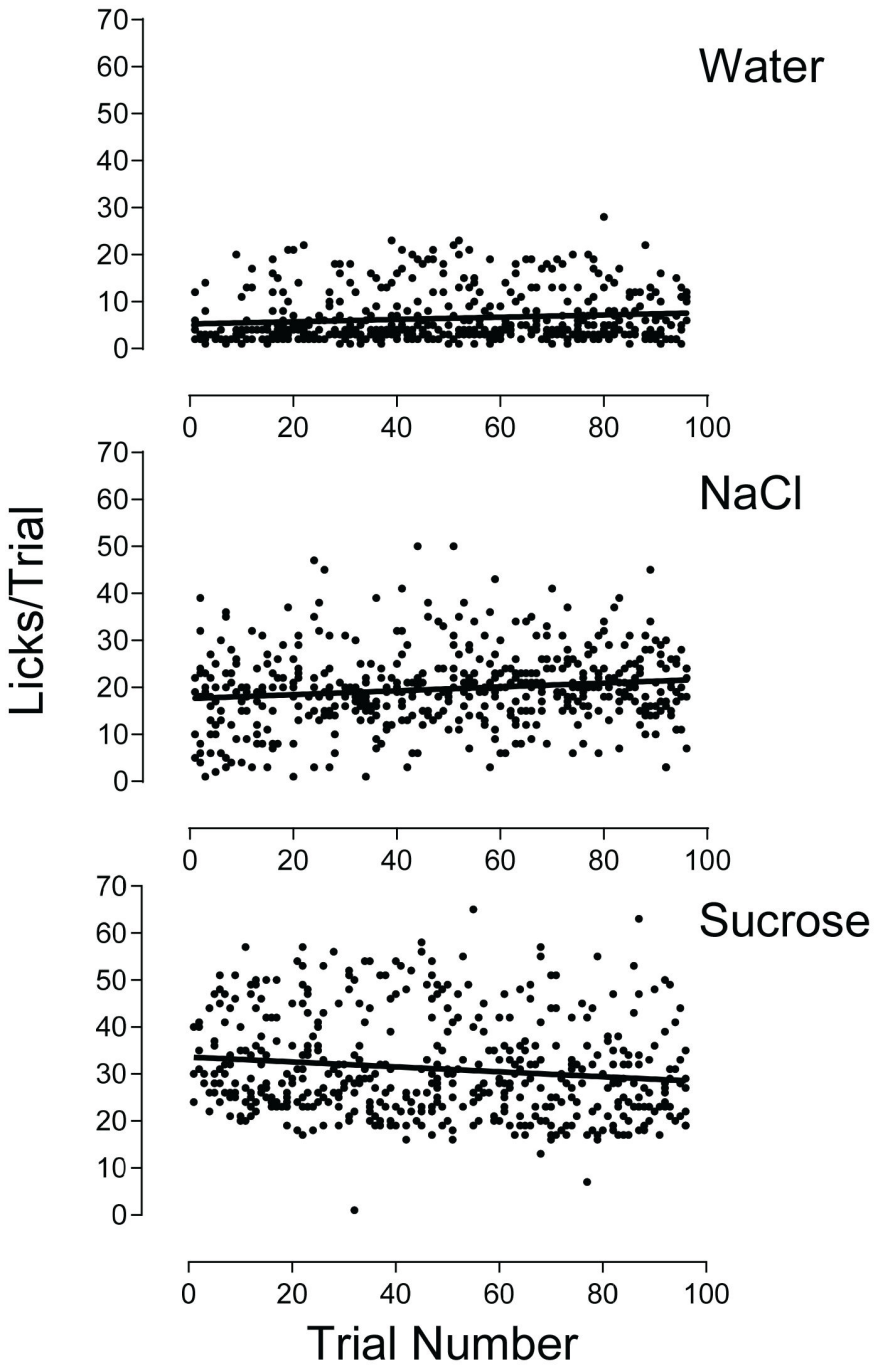
Relationship Between Lick Rates and Trial Number for Water, Sucrose, and NaCl. Data points are licks per trial plotted according to their corresponding trial number. All data in the plots were generated by 4 rats across four training sessions in which water, 100 mM sucrose, and 100 mM NaCl each were presented in 32 trials per session (thus, 4 rats x 32 trials x 4 sessions = 512 data points for each tastant.) The lines within the plots represent the results of the linear regression.

In an effort to determine whether the consumption of nutritive tastants or food pellet reinforcers over the 96 trials in a session could have impacted the palatability measure, we examined lick rate data for water, sucrose, and NaCl (tastants with nutritional significance) as a function of trial number on which they were presented. Data on licks per trial were collected from the four training sessions just prior to the introduction of citric acid as a test article (i.e., in the training sessions that occurred between the tests shown in Figure 2A and 2B). These sessions were chosen because the density of trials for each of the three tastants was greatest among all of the plate configurations used for these studies. Accordingly, water, 100 mM sucrose, and 100 mM NaCl each were allocated to 32 wells of the 96-well plate and presented in randomized order. The numbers of licks per trial for all four rats across the four sessions were retrieved and plotted as a function of trial number for each of the tastants (Figure 5). A linear relationship between lick rate and trial would be expected if palatability was affected by the cumulative intake of nutrients (from food pellets and tastant sample) incrementally occurring throughout the session. Analysis by linear regression indicated a slight, but significant, linear function (slope = -0.05, p = 0.0027) associating lick rate and trial number for sucrose. The negative slope of the function indicated that higher lick rates for sucrose tended to occur early in the sessions and gradually declined—but only slightly—toward the end. From the linear function, the sucrose lick rate dropped approximately 15% (from 34 to 29 licks per trial, first and last trials respectively.) Similarly, a weak functional relationship between lick rates and trial number was found for both water (slope = 0.02, p = 0.0073) and 100 mM NaCl (slope = 0.04, p = 0.0019). The positive slopes for the functions indicated that lick rates tended to gradually increase as the sessions progressed, but as was the case for sucrose, the change was slight. Since most of the variability in the data did not result from the relationships between lick rate and trial number (sucrose: R^2^ = 0.02; water: R^2^ = 0.01; NaCl:R^^2^^ = 0.02), it can be reasonably inferred that the impact of nutrient consumption during the sessions on the palatability measure was minimal.

### Capacity for Multiple Concentration–response Functions within a Single Test Session

With an assay in place for efficient evaluation of sweet taste, we then established and analyzed the concentration–response functions of a variety of other sweeteners. With many wells available, we were able to test several sweeteners at a time with multiple replicates at each concentration, improving precision of curves fit to the data by non-linear regression. Regression analysis of the concentration–response functions yielded EC50 values and curve maxima (representing efficacy relative to responses for sucrose), generally with narrow confidence intervals (Table 1).

**Table 1:** Potencies and Efficacies of Sweeteners.

	**EC50 (CI95%)**		**%Sucrose Maximum**	
**Sweetener**	**Taste Quality**	**Palatability**	**Taste Quality**	**Palatability**
Sucrose	28 (13-61)	53 (23-123)	100	100
Fructose	204 (101-412)	215 (112-468)	100	100
Glucose	290 (175-480)	265 (153-501)	100	100
Trehalose	>800	>800	ND	ND
Maltose	41 (13-136)	63 (36-101)	89	97
Sucralose	9 (3-32)	5 (wide)	75	55
Saccharin	2 (1-3)	1 (1-3)	82	85
Ace K	3 (2-5)	2 (1-4)	90	94
Glycyrrhizic Acid	6 (1-27)	5 (2-9)	56	86
Rebaudioside A	0.2 (0.1-0.4)	0.3 (0.1-0.5)	83	79
Stevioside	0.3 (0.1-0.7)	0.3 (0.1-0.9)	78	75
L-Glycine	75 (55-91)	63 (22-104)	95	97
SC 45647	0.01 (0.01-0.03)	0.01 (0.01-0.04)	100	100

Values were derived by non-linear regression of concentration–response data obtained from tests that each was limited to two test article sweeteners (in addition to sucrose). Potencies are given as EC50 in mM with 95% confidence intervals in parenthesis. Sweet-taste efficacy values are given as the maximum sucrose-appropriate lever responding relative to sucrose. Data for each sweetener are representative of data generated by at least two independent experiments. ND = not determined. “Wide” indicates that the curve fit did not yield an accurate confidence interval. See Figure S10 for plate configuration corresponding to the tests that generated the data for Table 1.

**Figure 6:**
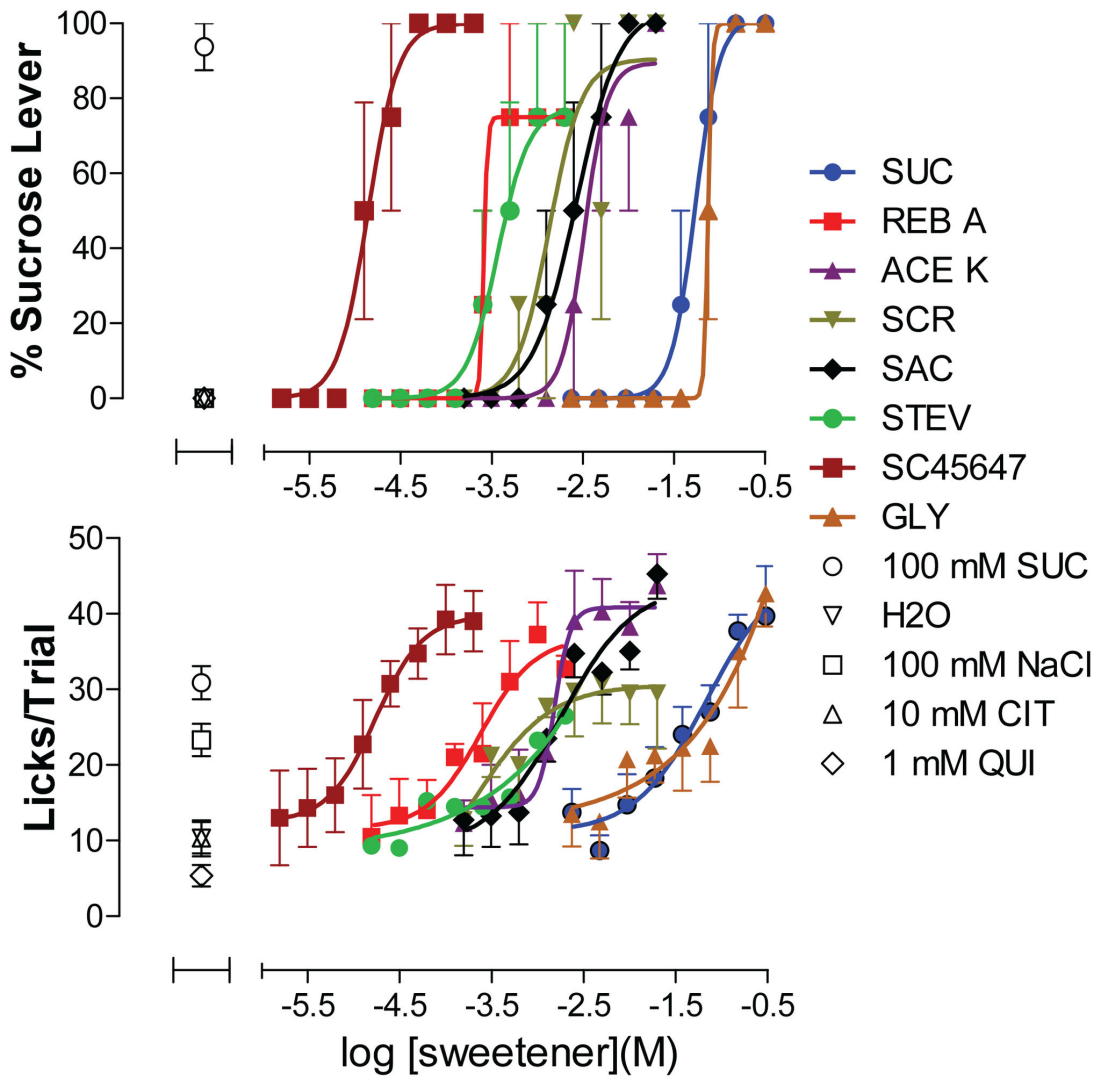
Concentration–response Functions for 8 Different Sweeteners in a Single Test Session. Eight sweeteners (SUC = sucrose, REB A = rebaudioside A, ACE K = acesulfame potassium, SCR = sucralose, SAC = saccharine, STEV = stevioside, GLY = L-glycine, and SC45647) were tested for both taste quality and palatability across a range of concentration. Positive control: 100 mM SUC = sucrose; Negative controls: 10 mM CIT = citrate, 1 mM QUI = quinine, water and 100 mM NaCl. Upper panels shows graphs of sweet taste quality plotted as percent of responses made on the sucrose lever. Lower panel shows concentration–response functions for palatability, plotted as mean licks/trial, obtained in the same experiment. Each concentration of test article was dispensed into a single well of a 96-well plate. Data are plotted as mean of responses to contents from a single well per concentration of test article and 6-7 wells for controls per rat, averaged across 4 rats. Error bars are SEM. Data are representative of 3 equivalent experiments. See Figure S5 for plate configuration corresponding to the experiment illustrated in Figure 6.

The capacity for carrying out multiple concentration–response testing within a single session for a single cohort of rats was next tested. A panel of 8 compounds (including sucrose) known to be sweet to both humans and rats was dispensed at varying concentrations, each in a single well of a 96-well plate. The plate was replicated for each of the 4 rats (see Figure S5). Concentration–response functions were discerned for all 8 sweeteners with potencies ranging approximately 4 orders of magnitude (Figure 6). Even with a minimum of replicates, concentration–response functions were consistent with EC_50_ values and curve maxima obtained from experiments with more replicates per concentration (given in Table 1).

### Potential for In Vivo Primary Screen of Sweet Taste Properties

**Figure 7:**
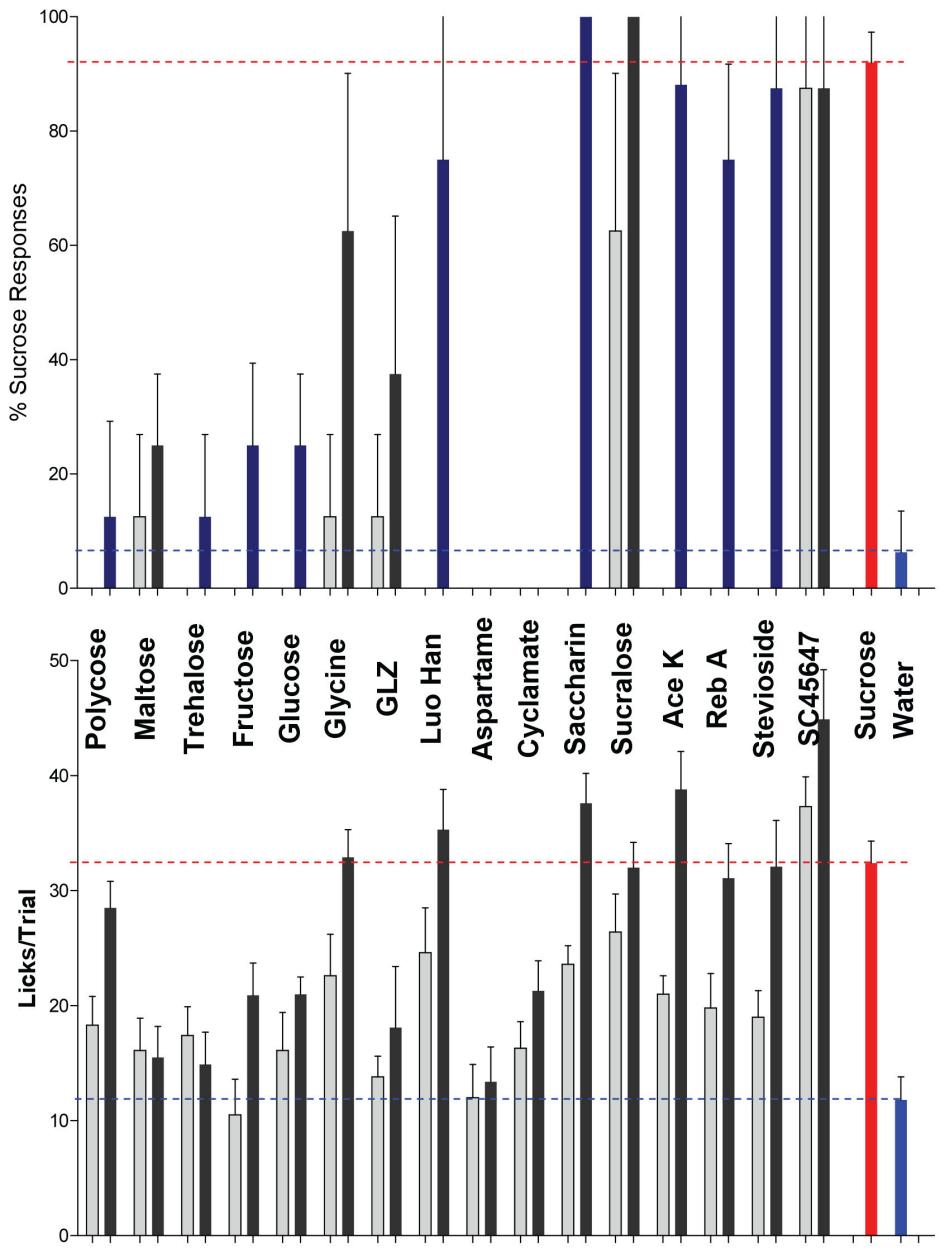
Test of the Potential for an In Vivo Primary Screen for Sweet Taste Properties. A panel of 15 sweeteners and polycose was tested by a cohort of 4 rats for detection of sweet taste quality (plotted as percent sucrose-appropriate lever presses, upper panel) and palatability (licks/trial, lower panel). Data for each sweetener (both taste quality and palatability) appear directly above and directly below their corresponding labels. Luo Han = Luo han guo, GLZ = glycyrrhizic acid, Reb A = rebaudioside A). Dashed blue and red lines respectively mark the mean responses to water (negative control) and to 100 mM sucrose (positive control). A single low (light gray bars) and single high concentration (dark gray bars) of each test sweetener was dispensed in two wells, and controls in 6-7 wells each, per plate. Concentration pairs were 0.01 and 0.1 mM (SC45647, stevioside, rebaudioside A), 0.1 and 1 mM (saccharin, Ace-K, sucralose), 1 and 10 mM (aspartame, cyclamate, glycyrrhizic acid) and 10 and 100 mM (L-glycine, glucose, maltose, fructose, trehalose). Luo han guo was tested at 1 and 10 mg/ml and polycose at 1% and 10% solutions. Results were averaged across 4 rats and the data shown in the figure are representative of two equivalent experiments. See Figure S6 for plate configuration corresponding to the experiment illustrated in Figure 7.

A panel of tastants comprised of 15 known sweeteners and polycose was assembled to test the potential of the methodology for performing a primary screen for novel sweeteners. One challenge facing the establishment of an in vivo primary screen for sweeteners is choosing the proper concentrations at which to test, since potencies ranging at least 4 orders of magnitude are displayed by known sweeteners (Figure 6). A strategy of screening at a single high concentration might result in the rejection of compounds that have aversive properties at high concentrations but favorable properties at lower concentrations. Conversely, screening at a single low concentration potentially will miss sweeteners active at higher concentrations. The strategy therefore should aim for a desired concentration range for the application endpoint in mind. Therefore, two concentrations representing a high and low concentration (separated by 10-fold) at which the sweeteners in the panel are known to be active were chosen to illustrate the principle. The ranges chosen were 10 and 100 mM, 1 and 10 mM, 0.1 and 1 mM, which represent three different potency-based strategies for discovery of novel sweeteners (luo han guo, which is a mixture, was tested at 10 mg/ml and 1 mg/ml; polycose, a mixture of glucose polymers, was tested as 1 and 10% solutions). As seen in Figure 7, many of the sweeteners were identified at one concentration but not the other. Polycose, which is sensed as an appetitive tastant in rodents but not through stimulation of sweet receptors [30], was not identified as sweet in the simulated screen, but clearly was appetitive as evinced by elevated lick rates. Rats and mice are known to be insensitive to the taste of aspartame and cyclamate [31,32], and neither compound elicited any sucrose-appropriate lever presses during the simulated screen. An elevation in licking relative to that for water was observed for cyclamate, but is likely to have been due to the presence of molar equivalents of sodium. Interestingly, the carbohydrates glucose and fructose elicited only low levels of responding in either taste quality or palatability. Full concentration–response functions obtained for these sweeteners confirmed that they are active at still higher concentration ranges (Table 1).

### Capacity for Evaluating Modulators of Sweet Taste

The ability to perform multiple concentration–response functions within a single session provides a powerful way to study the effects of compounds that act through allosteric or other mechanisms to modify sweet taste. Two putative sweet-taste inhibitors, alloxan [33] and ZnSO_4_ [34,35], were tested for their ability to shift the sucrose concentration–response function.

**Figure 8:**
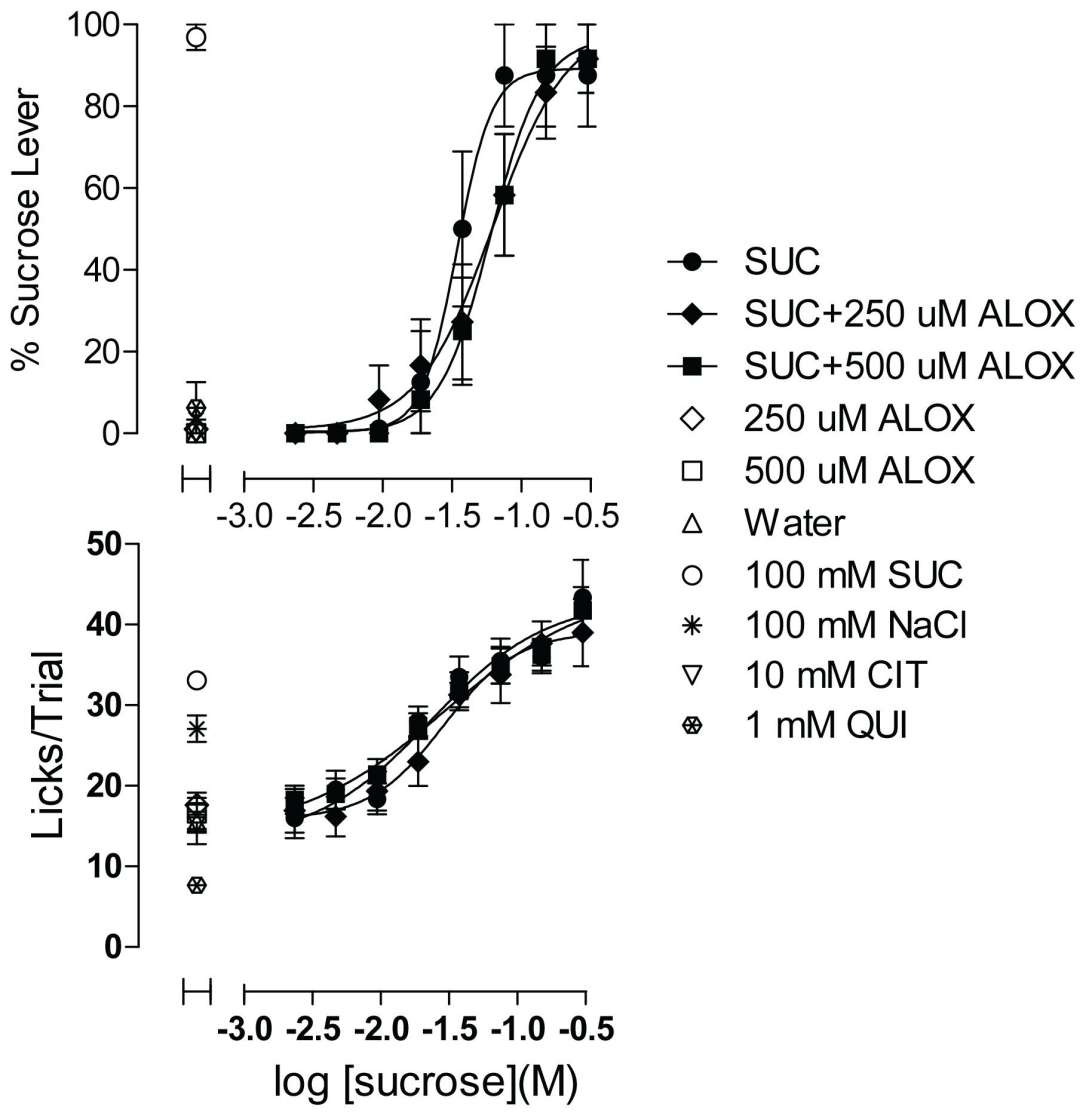
Effect of Alloxan on Sucrose Concentration–response Function. Each concentration of sucrose plus alloxan was dispensed in 3 wells, and of sucrose alone in 2 wells, per plate; controls were dispensed in 4-6 wells each per plate (See Figure S7 for plate configurations corresponding to the experiment illustrated in Figure 8). Upper panel shows sweet taste quality plotted as percent of responses made on the sucrose lever. Lower panel shows concentration–response function for palatability plotted as mean licks per trial. Data are plotted as mean of responses averaged across 4 rats. Error bars are SEM. SUC = sucrose, ALOX = alloxan, CIT = citrate, QUI = quinine. Data are representative of 3 equivalent experiments.

A modest rightward shift was observed in the sucrose function when 250 µM alloxan was added to all of the sucrose concentrations (Figure 8). Doubling the concentration of alloxan produced no further effect. In either case, though the modest shift was observed consistently across experiments, the changes in EC50 or Hill slope parameters did not reach statistical significance (Extra sum-of-squares F test). The apparent shift was evident only in the taste quality function; alloxan at either 250 or 500 µM had no discernable impact on the palatability function of sucrose (Figure 8). Both concentrations of alloxan also were tested independently of sucrose in the same session for any sign of intrinsic taste properties. Alloxan did not elicit any sucrose-appropriate lever pressing and lick rates on alloxan trials were indistinguishable from those for water (Figure 8).

**Figure 9:**
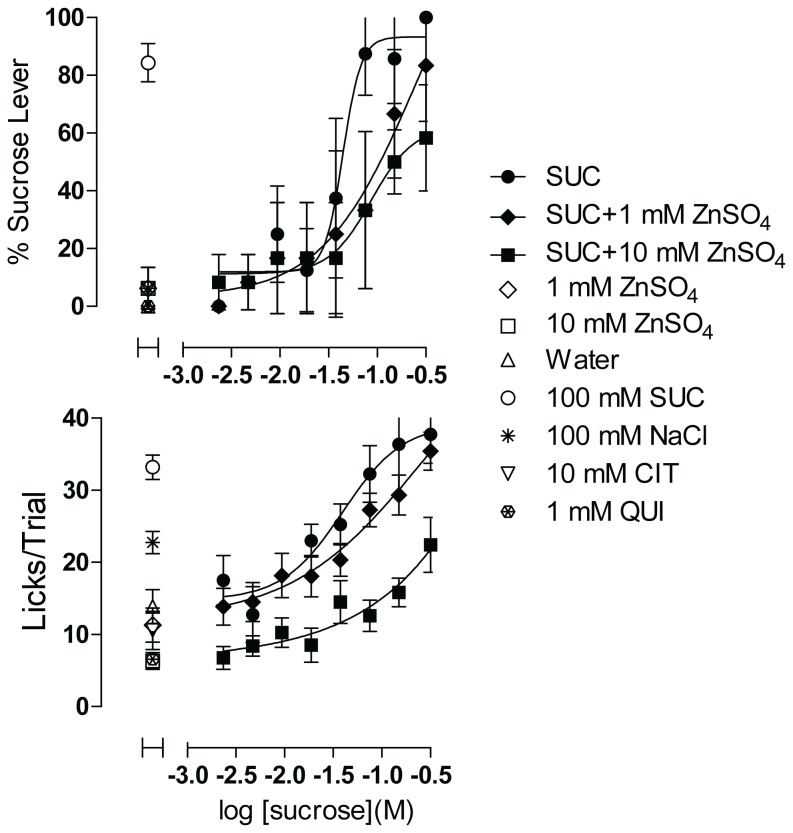
Effect of ZnSO_4_ on Sucrose Concentration–response Function. Each concentration of sucrose plus ZnSO_4_ was dispensed in 3 wells, and of sucrose alone in 2 wells, per plate; controls were dispensed in 4-6 wells each per plate (See Figure S7 for plate configuration corresponding to the experiment illustrated in Figure 9). Upper panel shows sweet taste quality plotted as percent of responses made on the sucrose lever. Lower panel shows concentration–response function for palatability plotted as mean licks per trial. Data are plotted as mean of responses averaged across 4 rats. Error bars are SEM. SUC = sucrose, CIT = citrate, QUI = quinine. Data are representative of 3 equivalent experiments.

When added to the sucrose concentration range, 25 mM ZnSO_4_ (results not shown) completely suppressed sucrose-appropriate lever pressing and lick rates for sucrose, but also imparted an aversive taste on its own when tested independently of sucrose, as indicated by lick rates that were equivalent to those of 1 mM quinine. The concentration–response function for sucrose alone and the positive control of 100 mM sucrose were negatively impacted as well, indicating that the taste effects of ZnSO_4_ carried over across trials to affect responses throughout the session. Reducing the concentrations of ZnSO_4_ to 1 and 10 mM enabled evaluation in subsequent test sessions. ZnSO_4_ at both concentrations appeared to have affected sucrose taste responses (Figure 9), though statistically significant differences (Extra sum-of-squares F test) among the concentration–response functions were not detected for either taste quality or palatability. Adding 10 mM ZnSO_4_ to all concentrations of sucrose resulted in a downward displacement of the palatability function, suggesting that lick rates were suppressed evenly at all concentrations of sucrose. As evident in the lick rates for both concentrations tested alone, ZnSO_4_ most likely influenced sucrose taste responses by adding an aversive intrinsic taste. ZnSO_4_ at 10 mM was as aversive as 1 mM quinine, both tastants significantly suppressing lick rates relative to those elicited by water (F(3,62)=4.885, p=0.004; 1 mM ZnSO_4_ vs water not significant, 1 mM ZnSO_4_ vs 10 mM ZnSO_4_ not significant, 10 mM ZnSO_4_ vs water p < 0.01, 1 mM quinine vs water p < 0.01). Thus alloxan (if at all effective) and ZnSO_4_ can potentially modify taste responses to sucrose but are not likely to do so by inhibition of sweet taste signaling at the concentrations tested.

### Method Applied to Salt and Umami Tastes

**Figure 10:**
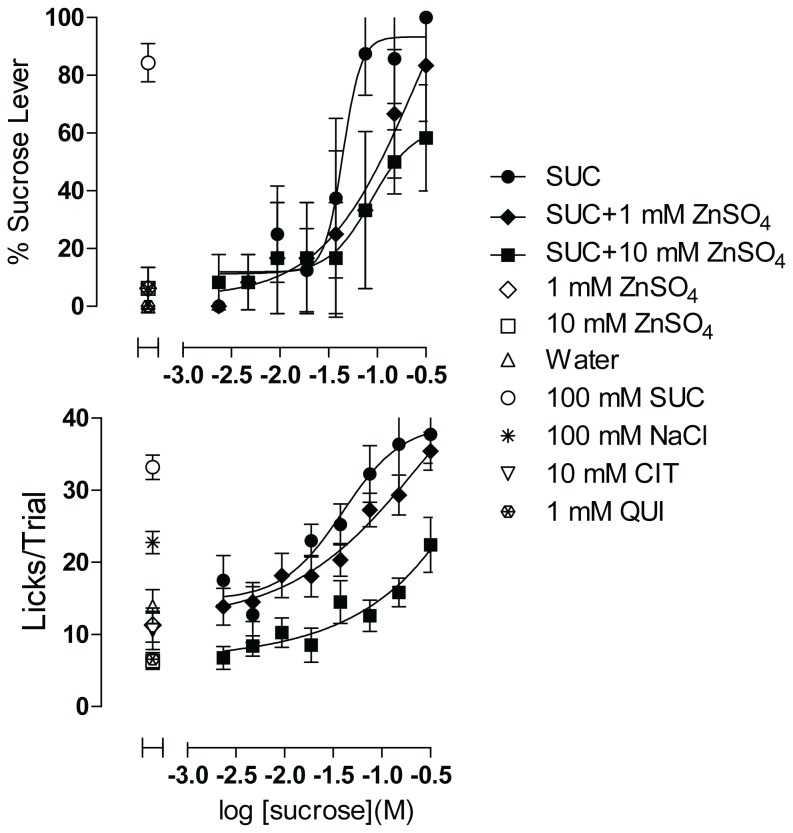
Concentration–response Functions for Salt and Umami Tastes. Upper graphs in each panel show taste quality plotted as percent of responses made on the standard-appropriate lever. Lower panels show concentration–response functions for palatability plotted as mean licks per trial. Data are plotted as mean of responses to contents from 4 wells per concentration of test article and 10-14 wells for controls per rat, averaged across 4 rats. Error bars are SEM. **A**: Rats were trained to discriminate the taste of 100 mM NaCl (○) from water (▽), 100 mM sucrose (□), 10 mM citric acid (△), and 1 mM quinine (◇). Test article: ● = NaCl. **B**: Rats were trained to discriminate the taste of 100 mM MSG+100 µM amiloride (○) from water (▽), 100 mM sucrose (*), 100 mM NaCl (□), 10 mM citric acid (△) and 1 mM quinine (◇). Test article: ● = MSG + amiloride. See Figure S8 for plate configurations corresponding to the experiment illustrated in Figure 10A; Figure S9 corresponds to the experiment of Figure 10B.

We applied the same methodological approach to establishing taste discriminations for two additional appetitive tastes, salt and umami. A cohort of 3 rats was trained to distinguish the taste of 100 mM NaCl from water, quinine, citric acid and sucrose (Figure 10A). Following the protocol worked out for sucrose discrimination where non-standard taste controls were sequentially added, the discrimination of NaCl was established to 90% salt-appropriate responding in all rats by day 24 of discrimination training (mean = 18, SEM = 2). Lick rates for NaCl were essentially maximal at 100 mM (mean licks/trial = 24, SEM = 1) and were significantly higher than for water (mean licks/trial = 12, SEM = 2) and apparently (but not statistically) less than for 100 mM sucrose (mean licks/trial = 29, SEM = 2; F(2,106)=26.34, p<0.0001, NaCl vs. water p <0.001, sucrose vs. water p<0.001, NaCl vs. sucrose not significant.)

To establish a discrimination for umami taste, the training cue was composed of 100 mM MSG with 100 µM amiloride added to minimize sodium taste [36] associated with the MSG. Since some taste properties of MSG and sucrose have been shown to overlap for rats [16] it was important to demonstrate that the taste of MSG could be distinguished from sucrose by the rats for developing an effective umami discrimination assay. Thus, water, citric acid, NaCl, quinine, and 100 mM sucrose were sequentially added to the discrimination training (sucrose added last). Achieving 90% umami-appropriate lever presses for a cohort of 4 rats took substantially longer than was the case for the salt taste discrimination training (mean = 39 days, SEM = 8). Nevertheless, all rats within the cohort eventually were able to accurately discriminate the MSG/amiloride cue from the non-standard controls, including 100 mM sucrose, and umami concentration–response functions for taste quality and palatability were established (Figure 10B). Lick rates for the MSG/amiloride training cue (mean = 29, SEM = 4) were equivalent to those for the 100 mM sucrose control (mean = 31, SEM = 3), and significantly higher than for water (mean = 10, SEM = 3; F(2,130)=22.64, p <0.0001, MSG/amiloride vs. sucrose not significant, MSG/amiloride vs. water p <0.001, sucrose vs. water p <0.001). Lick rates were maximal (mean = 33, SEM = 3) at 300 mM MSG, the highest concentration tested.

## Discussion

A number of mammalian animal models that are effective for determining taste quality and palatability have been in use for decades. Testing for palatability has best been accomplished by brief-access assays that use the Davis Rig [37], an automated lickometer that can present up to 16 sipper tubes to a rodent. Full concentration–response functions for lick rates can be obtained within a 30-minute test session using this method [14,15,22,38]. Since subjects usually are water-deprived, basal lick rates for water are relatively high making assessment of appetitive solutions difficult in brief-access assays [22,39].

Recent innovative methods for measuring taste quality have been reported that offer some advantage over existing models. For example, a novel method recently has been described in which rats are trained to associate each basic taste with a spatial location [40]. The principle advantage of this method is that sensory properties of test samples can be evaluated across taste modalities in a open field, making it an ideal approach not only for understanding complexities in taste quality but also for associating behavioral responses to concurrent neurophysiological recordings.

Grobe and Spector [8] have developed an operant taste discrimination paradigm in which rats are trained to discriminate the taste of a standard cue from the remaining basic tastes for the purpose of constructing taste quality profiles for novel tastants and tastant mixtures. The method we describe here builds on a similar paradigm, but has increased testing capacity by incorporating microtiter plate technology used for pharmaceutical drug screening into operant taste discrimination and palatability measurement.

As we have shown, rats were trained in an operant taste discrimination using a novel apparatus designed to randomly present small volumes of tastant solutions dispensed in standard 96-well plates. Cohorts of 3 to 4 rats were able to evaluate all 96 samples within approximately 90 minutes, and were able to identify the taste of sucrose (as well as the tastes of NaCl and MSG) from other basic tastes with an accuracy approaching 100% with no sign of fatigue or desensitization. Since the samples were dispensed in wells and were presented one at a time, cross-contamination of tastants was essentially eliminated. The possibility of carry-over taste effects across the 96 trials was minimized by consumption of 45 mg grain-based pellets in the interval between trials.

The taste quality measurement of our method was operationally defined as the discrimination of a single concentration of a standard tastant (e.g., 100 mM sucrose) from tastant solutions, also at single concentrations, that represent the remaining basic tastes. Training a single concentration of taste stimulus as the discriminatory cue runs the risk of inadvertently establishing a discrimination based on stimulus intensity (or some other non-gustatory property), rather than taste quality. To minimize this risk, other methods (see for example 8] [20) base the discrimination task on multiple concentrations of tastant, presuming to neutralize the effects of stimulus intensity and thereby enhancing the relative salience of taste quality among other possible sensory cues. This strategy is particularly relevant when the experimental design is limited to a comparison between two different taste stimuli. In our method, taste intensity was a significant contributor to the sensory cue in the earliest stages of discrimination training when the choice was between 100 mM sucrose and water. The discriminatory cue then shifted completely to taste quality soon after the addition of a second tastant, 100 mM NaCl, as a non-sucrose control in the training procedure. Thereafter, all non-sweet tastants at multiple concentrations were discriminated from sucrose. Basing the discrimination task on a cue composed of a single concentration of tastant allows taste quality to be analyzed as a concentration–response function in a manner analogous to the way in which interoception of psychoactive drug effects (another class of receptor-mediated sensory phenomena) is studied using the drug-discrimination paradigm (for review, see 41] [42,.)

Making the appearance of the levers contingent upon licking also guaranteed that the sample had to be tasted in order for the discrimination task to be performed. Thus the discrimination could not have resulted from olfactory cues exclusively. Olfactory cues still could have played a role in the discrimination by means of retronasal olfaction [43], as must be the case for all taste tests that do not render the animal anosmic.

Because of behavioral chaining, lick rates could be measured on the same trial as the discrimination choice. Interestingly, only one lick was required to produce the levers that operated the pellet dispenser, yet the rats continued licking more than was necessary. Chaining the licking to food-reinforced lever presses proved to be a good condition for increasing the observational window for measuring appetitiveness as well as aversiveness of taste solutions. The number of licks per trial was a function of the tastant, with intrinsically appetitive tastants (e.g., sucrose) eliciting high lick rates, and aversive tastes (e.g., quinine) low rates, relative to those recorded for water. Not surprisingly, the volume consumed per trial was a function of the number of licks. But with the many data points that were readily available from just three sessions, the function relating licking to consumption could be rigorously defined for individual rats. The nonlinear functions obtained are reflective of the mechanics of withdrawing fluid by lapping from a well with restricted access. It was possible to determine from the function the volume withdrawn from the well by each lick. The extrapolated volumes of the first licks (6 µl and 8 µl for Rats 18 and 21, respectively; Figure 4) are consistent with previous exhaustive literature on the topography of rat licking (for review and detailed discussion on the mechanics of licking from spouts and unrestricted lapping see reference [44]). Knowing the concentration, the lick-volume functions can further be used to quickly ascertain an estimate of the total amount of tastant consumed during the session. For example, using the function for Rat 18 (Figure 4) as a guide, the mean licks/trial values used to plot the palatability graph in Week 1 of Figure 3 can be located on the function and summated to obtain a total amount of 115 mg of sucrose consumed during the session; the caloric equivalent of three 45 mg food pellet reinforcers (0.45 kcal).

Lick rate as a measure of palatability is subject to influence by motivational states of nutritional need [45] [46]. Given that the rats of our method ingest nutritive tastants and 96 food pellet reinforcers during a session it was important to ascertain whether lick rates changed across the trials, which consequently could affect interpretation of the palatability measure. To this end, the relationship between the numbers of licks per trial and trial number was examined from four training session in which the only three stimuli were the nutritive tastants water, 100 mM sucrose, and 100 mM NaCl. These sessions were configured so that each tastant was randomly presented in 32 trials. Taking the data from all four rats in the cohort across the sessions, analysis by linear regression indicated that cumulative nutrient intake occurring over 96 trials had very little impact on the lick rates. Therefore, hedonic taste properties probably were the chief drivers of the relatively high lick rates for NaCl and sucrose (and possibly also MSG) observed in our assay.

The ability to obtain taste responses from 96 samples generated sufficient data to establish concentration–response functions for as many as 8 different sweeteners within a single session using only 4 rats. Concentration–response analysis provides a convenient means of comparing bioactive properties across multiple compounds and also is an effective way to explore functionality of biological systems. Relative orders of potency and efficacies can be discerned readily from visual inspection of concentration–response graphs, as is apparent in the results presented in Figure 6. Non-linear regression applied to in vivo concentration-response data provide quantitative measures of potency with precision indicated by attendant statistically determined confidence intervals. The 96-well format of our method is well-suited to rapid generation of concentration–response data for multiple tastant compounds at once, with low variability. Our results indicate that the rats were capable of detecting and reporting the taste of a variety of different kinds of sweeteners across a broad range in active concentrations ranges.

The synthetic compound SC45647 was the most potent tested (approximately 10 µM) and compounds such as the carbohydrate trehalose were the least potent with EC_50_ values well above that of sucrose. Differences in sweetness efficacy (i.e., maximal sweetness) were readily apparent from the asymptotes in the concentration–response functions among the sweeteners. The synthetic sweeteners SC45647 and acesulfame potassium elicited close to 100% sucrose-lever responding and palatability equivalent to sucrose, and therefore apparently were indistinguishable from sucrose to the rats in our study. Other sweeteners, such as the steviol glycosides rebaudioside A and stevioside consistently reached maxima of approximately 80% sucrose-appropriate lever pressing and elicited lower lick rates than for sucrose. Similar taste quality responses were observed for sucralose, but the lick rates were appreciably reduced relative to sucrose and other sweeteners, suggesting that the rats sensed sucrose-like sweetness but were also sensitive to other taste properties of sucralose that were aversive. Recent reports have indicated that individual rats can differ in their sensitivities to taste quality of sucralose and some other sweeteners [47,48]. Although not evident in the results from the rats used in the present study, further testing in our laboratory with new cohorts of rats suggests individual differences in the responses to sucralose, similar to those observed in the earlier reports. We anticipate an expansion of a systematic study of individual responsiveness to non-nutritive sweeteners using our methodology to rapidly screen rats for their response to sucralose and forming cohorts according to the responses to sucralose.

The ability to generate concentration–response functions with narrow confidence intervals provides additional advantage in that sensitive subtle changes on taste functions effected by taste modulators should be readily detectable. We examined the effects of two purported sweetness inhibitors, alloxan and ZnSO_4_, on the sucrose concentration–response function. An inhibitor that was specific to sweetness would be expected to shift the concentration–response function of sucrose to the right, or downward as a competitive or non-competitive antagonist. In effect, the inhibitor should make sucrose solution taste more like water. Alloxan had little impact on the taste quality function and none on the palatability function, whereas ZnSO_4_ affected both. Our functional analysis indicated that the effects of these compounds at the concentrations tested were not due to inhibition of sweet taste, but more likely resulted from the addition of intrinsic taste properties of the compounds to the sucrose solutions.

The assay can be scaled for in vivo primary screening of tastant libraries. Choosing the proper concentration at which to screen tastant libraries for taste activity is an opening challenge that mostly is determined by the goal for desired activity. As evident in Figure 7, some low potency sweeteners would not be detected if the screening concentration chosen aimed for detection of high potency sweeteners. The success of our simulated screen with 16 sweet compounds, each tested at a high and low concentration, suggests that as many as 32 compounds could be screened at a single concentration within a single session. Although the throughput is still lower than that of cell-based assays, screening smaller focused libraries would be practical using this methodology, and in the long run might be more productive since the hits would be not just receptor-active, but taste-active, providing additional information regarding taste quality and palatability.

Making a 96-well plate the basis for delivery of the taste stimuli has provided practical advantage by allowing an established commercial supply chain, created for the pharmaceutical industry, to be tapped. The dimensions of 96-well plates are standardized (with minimal variations) and are available from several vendors. Compound libraries typically are supplied in 96-well plates and their contents easily are transferred to test plates using hand-held multichannel pipettes or by automated liquid handlers. Because of the small volumes (50-300 µl) of the wells the amounts of tastant required for testing typically range well below 1 mg per sample, an especially important consideration when samples are precious, such as is often the case for natural products.

It is reasonable to ask whether the profile of taste sensation reported by a rat is sufficiently close to that of humans for their utility as subjects for discovery of commercially valuable sweeteners and taste modifiers. Rats are opportunistic omnivores and human pests, with an appetite for human food [49]. Table I shows that many compounds that are used as sweeteners by humans also are detected by rats as sweet, with potencies and efficacies similar to those reported by humans [50,51]. A few notable exceptions that have previously appeared in the literature were confirmed here (e.g., aspartame, cyclamate). Since rats are not humans it should be no surprise that their sensory profile is not equivalent to that of humans. However it is clear that there is considerable overlap between the two species, not only with respect to the many different kinds of sweeteners both species detect, but also in the concentration ranges in which the sweeteners are active. The results suggest that rats serve as a good approximation to what would be expected of human taste sensing, and it would be anticipated that the majority of compounds regarded as sweet by rats also will be sweet to humans.

Finally, we showed that the current methodology can be applied to the study of other appetitive taste systems by establishing concentration–response functions for taste quality and palatability for NaCl and an umami taste cue. It is reasonable to expect that the methodology can be extended to other tastes, whether basic or complex, and could be adapted for other species.

In summary, we have invented an apparatus and accompanying methodology based on the presentation of taste stimuli dispensed in 96-well plates to rats trained in an operant taste discrimination task. With this technology we have developed high throughput behavioral assays that simultaneously capture data on both taste quality and palatability, enabling rapid generation of concentration-responses functions for a multiplicity of tastants as well as the potential for primary in vivo screening of tastant libraries.

## Supporting Information

Video S1 **Close Up of a Rat Licking a Sample During a Single Trial.**
The video shows a close up of a trained rat proceeding through a single trial. The video begins a few seconds prior to the trial start. The rat anticipates the beginning of the trial, having likely attended to cues such as the sound of the x-y motion table moving the 96-well plate into place as well as passage of time during the inter-trial interval (30 seconds.) The aperture is set within a black plastic rectangular mounting located at the front edge of the chamber floor immediately in front of the pellet dispenser receptacle. Just prior to the trial start, the rat inspects the closed aperture. As soon as the tone sounds to signal the trial start, the trap door covering the aperture slides back to expose the 5 mm diameter aperture. (Illumination of the house light can be seen reflected off the chamber walls also as the trial begins; on the previous trial the rat made an incorrect choice on a control well consequently resulting in a 1 minute time-out.) The rat begins licking through the aperture for the contents of the well beneath the floor. Notice that the rat can insert only its tongue into the well. The first lick triggers the release of both levers from the front panel and the illumination of the stimulus lights over the levers (the right lever is not visible in this view and at this moment the left is blocked from view by the rat’s body.) The trial is a of the 100 mM sucrose training cue and the rat licks 32 times, each time breaking the path of the laser beam projected across the top of the well located 2 mm below the bottom surface of the cage floor. Disruptions of the laser path are detected by a photocell and are recorded by the computer program. When the rat is finished licking, it moves to the left (sucrose) lever to perform the lever-press operant (FR10.) The trial is completed upon the final lever press, the stimulus lights extinguish, the levers retract, and the door over the aperture closes. The correct lever choice was made thereby resulting in the delivery of a 45 mg food pellet, which the rat retrieves from the receptacle.
(WMV)

Video S2 **Rat’s Tongue Licking Viewed from Below the Chamber Floor as It Enters the Well.**
The perspective is from the front right corner of the subchamber, with the x-y motion table appearing prominently in the foreground. The top of the 96-well plate is visible just over the edge of the x-y motion table. A small red spot, the projection of the laser beam onto the photocell, is noticeable on the far left side in the 2 mm space between the top of the 96-well plate and the bottom of the cage floor (seen just to the right of center in this view.) The x-y motion table moves the plate to align a single well concentric with the aperture in the floor above. As the trial begins, the rat’s tongue appears through the aperture, illuminated by the laser beam. Notice that as the tongue reflects the laser’s light, the spot on the photocell momentarily disappears. The rat’s tongue rapidly moves into the well and withdraws, repeatedly breaking and restoring the laser beam path. Each disruption of the laser beam is detected by the photocell sending a signal to be recorded by the computer as a “lick.” This trial was of 300 mM sucrose; slow motion analysis of the video revealed 51 disruptions of the laser beam, which corresponded to the number of licks recorded by the computer.
(WMV)

Figure S1 **Plate configuration for Figure 2A.**
The figure shows a schematic diagram of the 96-well plate, and the contents of each well, used for the experiment. W = water, S=100 mM sucrose. Numeric values are the concentrations in mM of either NaCl or sucrose.
(TIF)

Figure S2 **Plate configuration for Figure 2B.**
The figure shows a schematic diagram of the 96-well plate, and the contents of each well, used for the experiment. W = water, N=100 mM NaCl, S = 100 mM sucrose. Numeric values are the concentrations in mM of either citric acid or sucrose.
(TIF)

Figure S3 **Plate configuration for Figure 2C.**
The figure shows a schematic diagram of the 96-well plate, and the contents of each well, used for the experiment. S=100 mM sucrose, N=100 mM NaCl, C = 10 mM citric acid, W = water. Numeric values are the concentrations in mM of either quinine or sucrose.
(TIF)

Figure S4 **Plate configuration for Figure 3.**
The figure shows a schematic diagram of the 96-well plate, and the contents of each well, used for the experiment. S=100 mM sucrose, Q=1 mM quinine, N=100 mM NaCl, C = 10 mM citric acid, W = water. Numeric values are the concentrations in mM of sucrose.
(TIF)

Figure S5 **Plate configuration for Figure 6.**
The figure shows a schematic diagram of the 96-well plate, and the contents of each well, used for the experiment. S=100 mM sucrose, Q=1 mM quinine, N=100 mM NaCl, C = 10 mM citric acid, W = water. Numeric values are the concentrations in mM of sucrose. See Figure 6 for concentrations of test sweeteners.
(TIF)

Figure S6 **Plate configuration for Figure 7.**
The figure shows a schematic diagram of the 96-well plate, and the contents of each well, used for the experiment. S=100 mM sucrose, Q=1 mM quinine, N=100 mM NaCl, C = 10 mM citric acid, W = water. Numeric values are the concentrations in mM of sucrose. See caption to Figure 7 for concentrations of test articles.
(TIF)

Figure S7 **Plate configuration for Figures 8 and 9.**
The figure shows a schematic diagram of the 96-well plate, and the contents of each well, used for the experiment. S=100 mM sucrose, Q=1 mM quinine, N=100 mM NaCl, C = 10 mM citric acid, W = water. Numeric values are the concentrations in mM of sucrose. See Figures 8 and 9 for concentrations of modifiers (alloxan and ZnSO_4_, respectively).
(TIF)

Figure S8 **Plate configuration for Figure 10A.**
The figure shows a schematic diagram of the 96-well plate, and the contents of each well, used for the experiment. S=100 mM sucrose, Q=1 mM quinine, N=100 mM NaCl, C = 10 mM citric acid, W = water. Numeric values are the concentrations in mM of NaCl.
(TIF)

Figure S9 **Plate configuration for Figure 10B.**
The figure shows a schematic diagram of the 96-well plate, and the contents of each well, used for the experiment. S=100 mM sucrose, Q=1 mM quinine, N=100 mM NaCl, C = 10 mM citric acid, W = water, M=100 mM MSG+100 µM amiloride. Numeric values are the concentrations in mM of MSG. 100 µM amiloride was added to each concentration of MSG in the concentration range.
(TIF)

Figure S10 **Plate configuration for Table 1.**
The figure shows the general template for the plate configuration used to generate data given in the table. S=100 mM sucrose, Q=1 mM quinine, N=100 mM NaCl, C = 10 mM citric acid, W = water. Numeric values are the concentrations in mM of sucrose. Concentration ranges for test sweeteners were obtained by successive 2-fold dilutions from the maximally effective concentrations of each.
(TIF)
