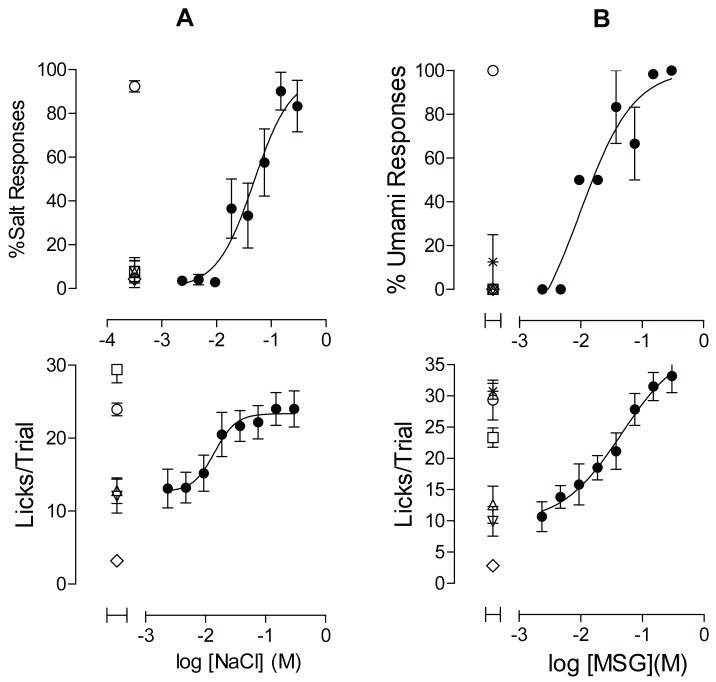# Correction: A High Throughput In Vivo Assay for Taste Quality and Palatability

**DOI:** 10.1371/annotation/6a76b80f-015c-4749-9b11-e3db355726e3

**Published:** 2014-01-10

**Authors:** R. Kyle Palmer, Daniel Long, Francis Brennan, Tulu Buber, Robert Bryant, F. Raymond Salemme

The image currently appearing as Figure 10 is incorrect. Please see the correct Figure 10 here: 

**Figure:**