# Supplementary figures and images for: A High Throughput In Vivo Assay for Taste Quality and Palatability
Source: PLoS One. 2013 Aug 12;8(8):e72391. doi: 10.1371/journal.pone.0072391 (PMC3741146; doi:10.1371/journal.pone.0072391)

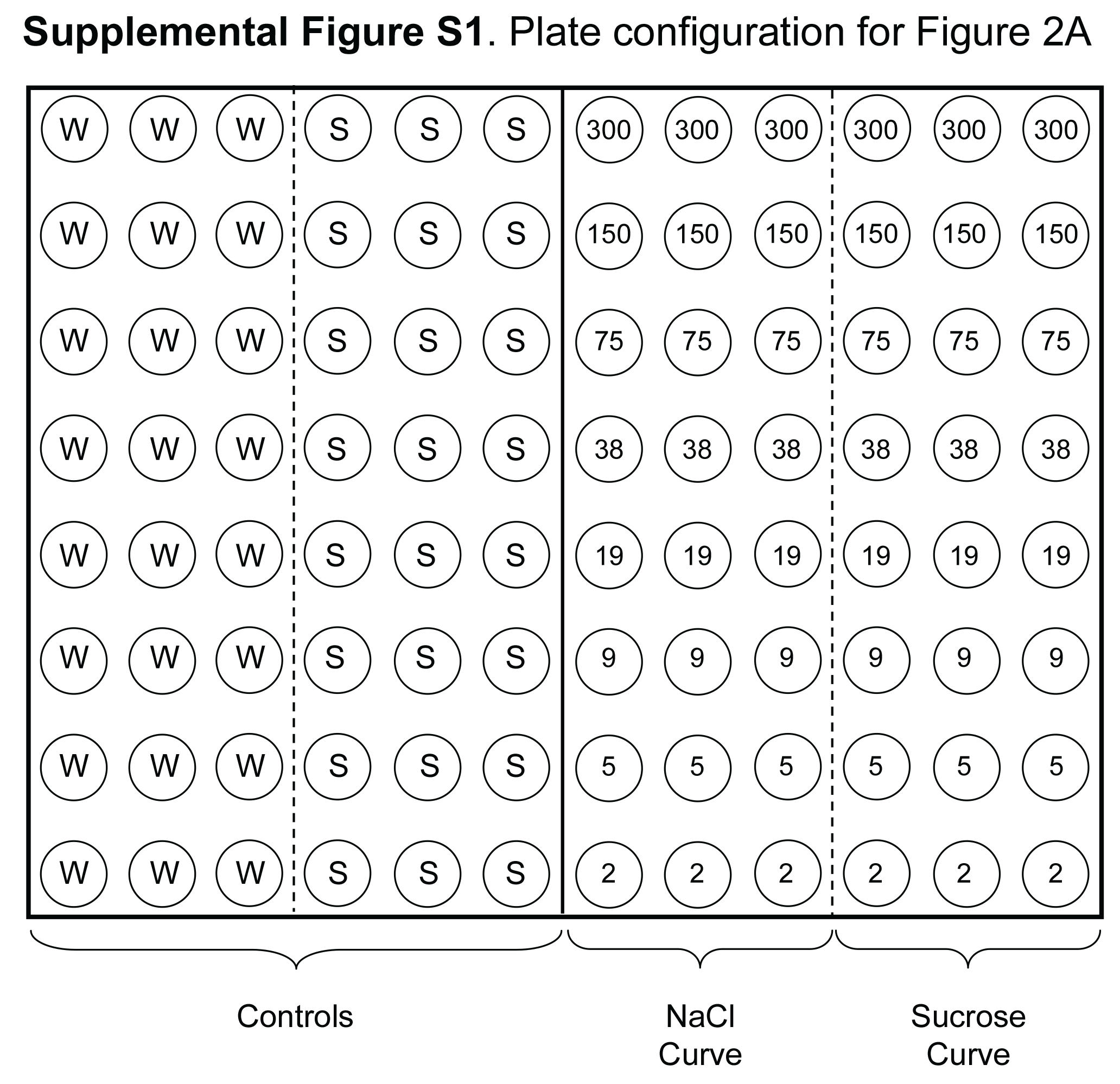

Supplement: Figure S1 — Plate configuration for Figure 2A. The figure shows a schematic diagram of the 96-well plate, and the contents of each well, used for the experiment. W = water, S=100 mM sucrose. Numeric values are the concentrations in mM of either NaCl or sucrose. (TIF) [file pone.0072391.s003.tif]

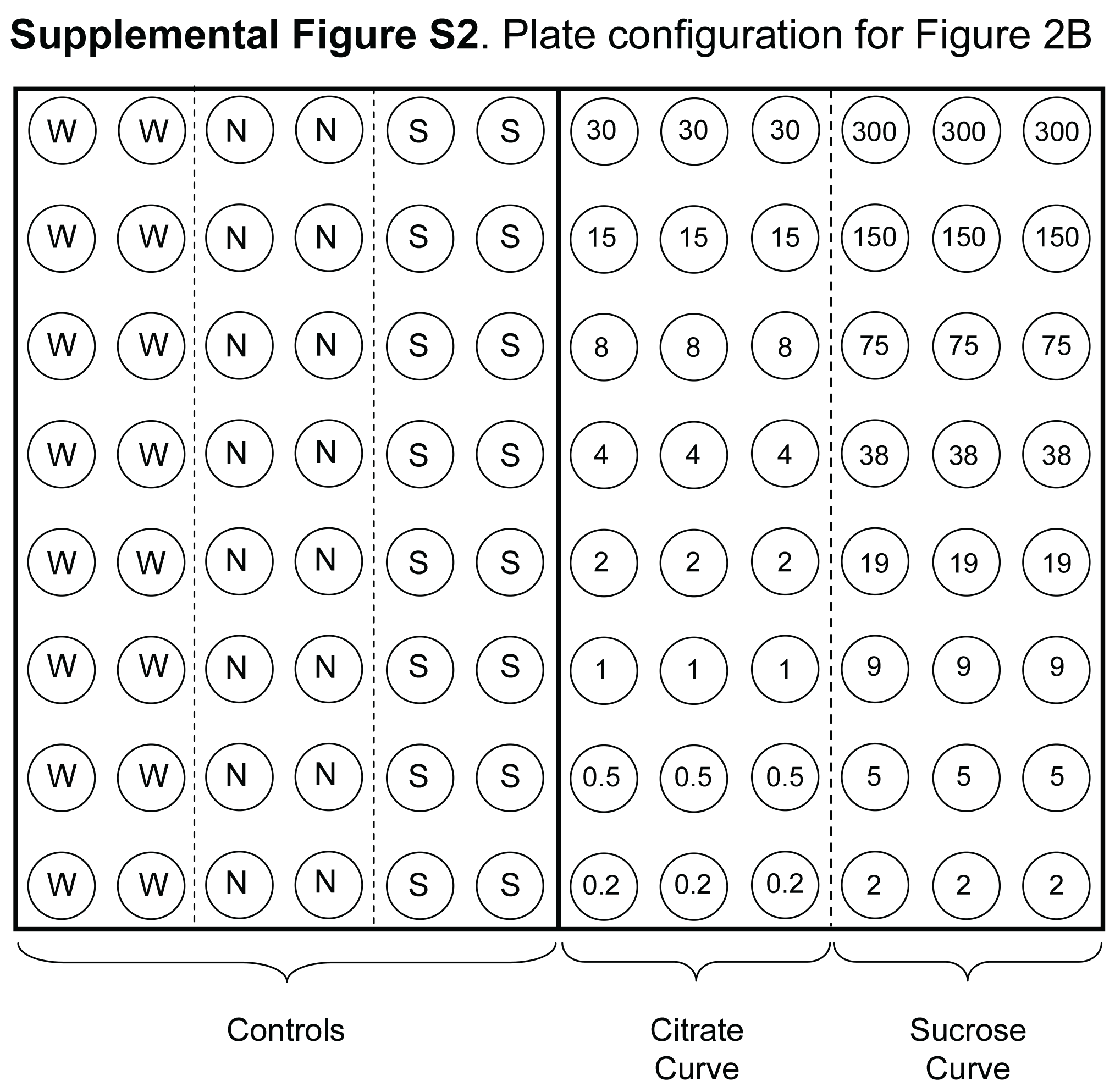

Supplement: Figure S2 — Plate configuration for Figure 2B. The figure shows a schematic diagram of the 96-well plate, and the contents of each well, used for the experiment. W = water, N=100 mM NaCl, S = 100 mM sucrose. Numeric values are the concentrations in mM of either citric acid or sucrose. (TIF) [file pone.0072391.s004.tif]

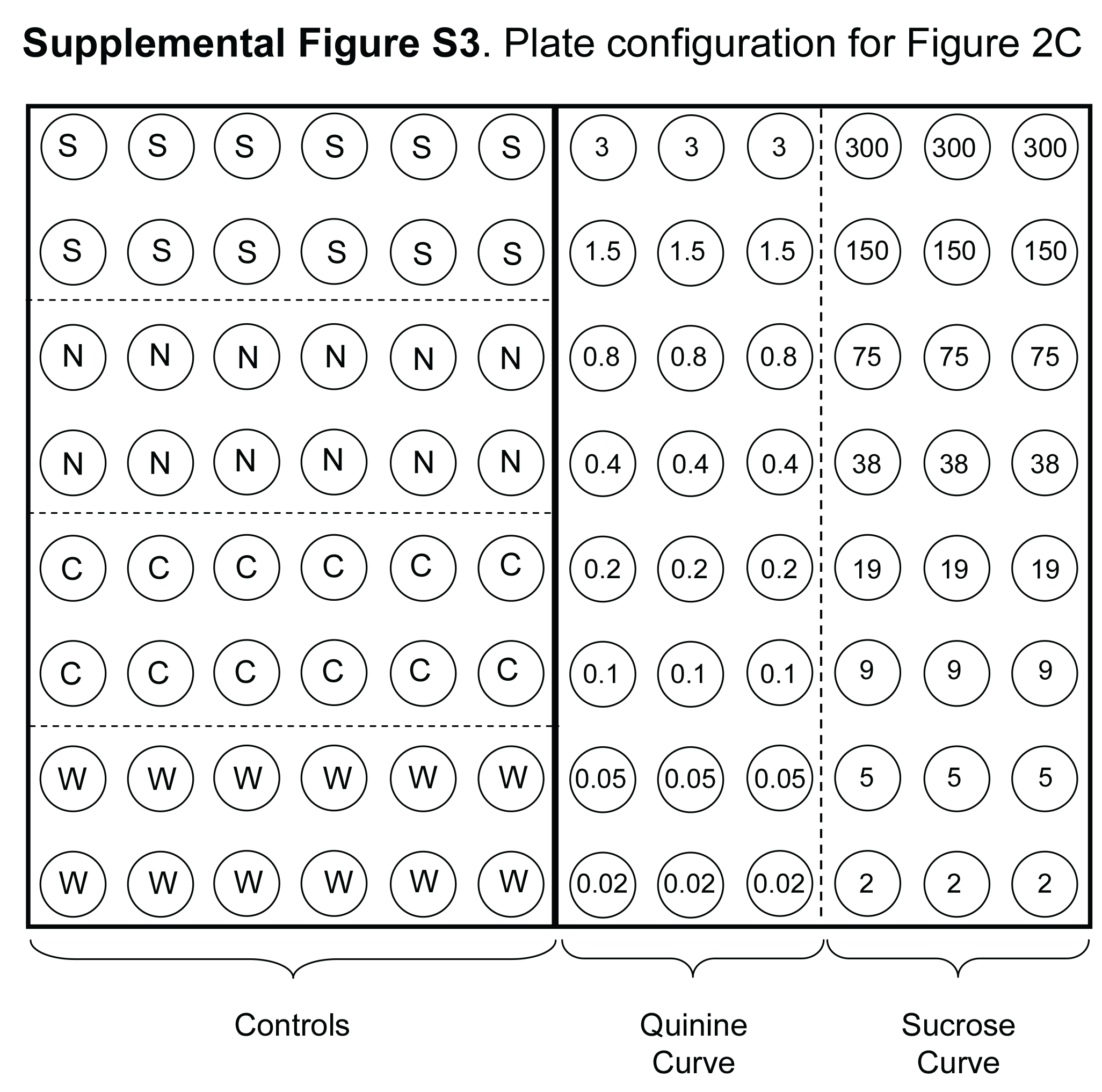

Supplement: Figure S3 — Plate configuration for Figure 2C. The figure shows a schematic diagram of the 96-well plate, and the contents of each well, used for the experiment. S=100 mM sucrose, N=100 mM NaCl, C = 10 mM citric acid, W = water. Numeric values are the concentrations in mM of either quinine or sucrose. (TIF) [file pone.0072391.s005.tif]

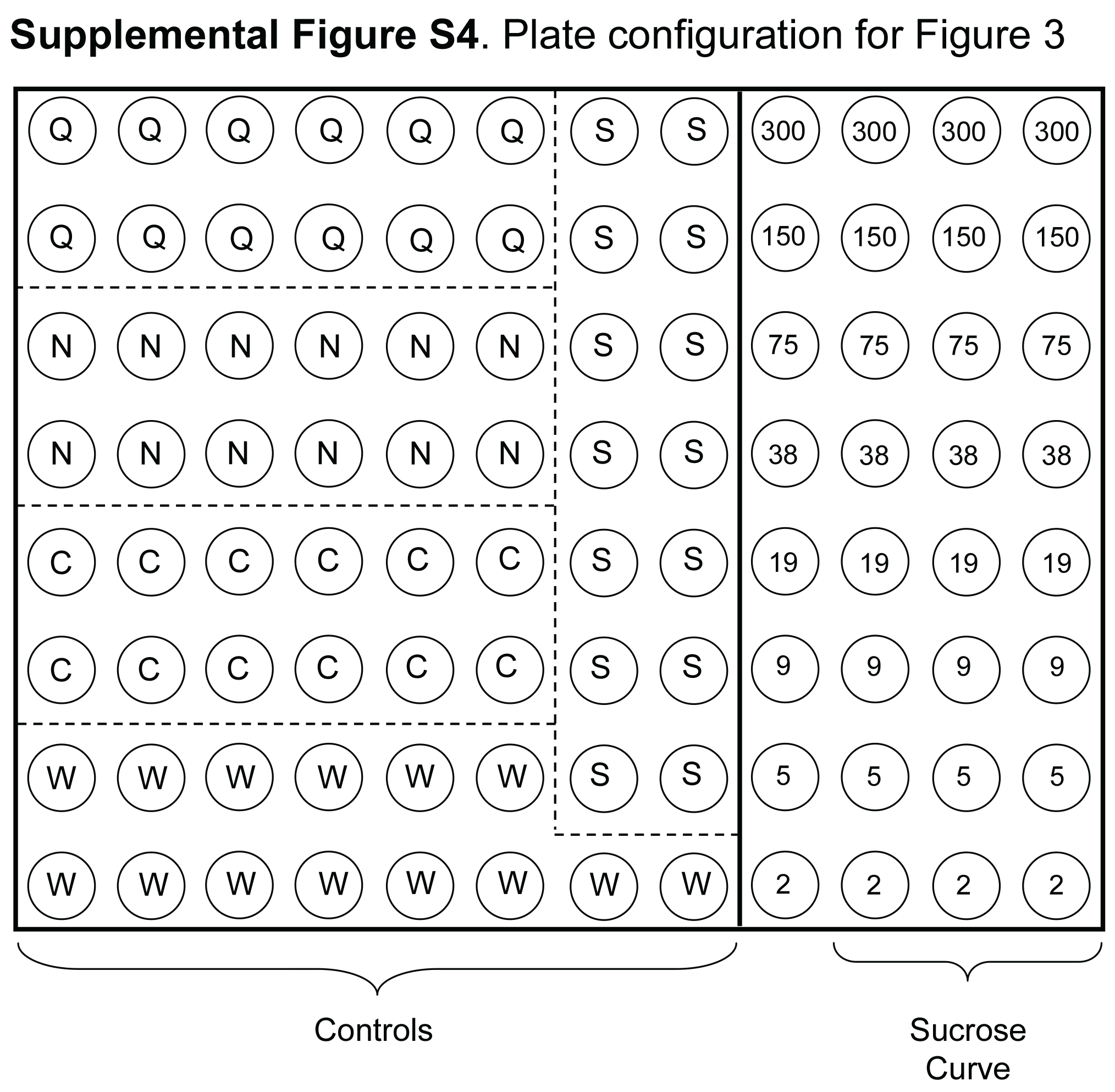

Supplement: Figure S4 — Plate configuration for Figure 3. The figure shows a schematic diagram of the 96-well plate, and the contents of each well, used for the experiment. S=100 mM sucrose, Q=1 mM quinine, N=100 mM NaCl, C = 10 mM citric acid, W = water. Numeric values are the concentrations in mM of sucrose. (TIF) [file pone.0072391.s006.tif]

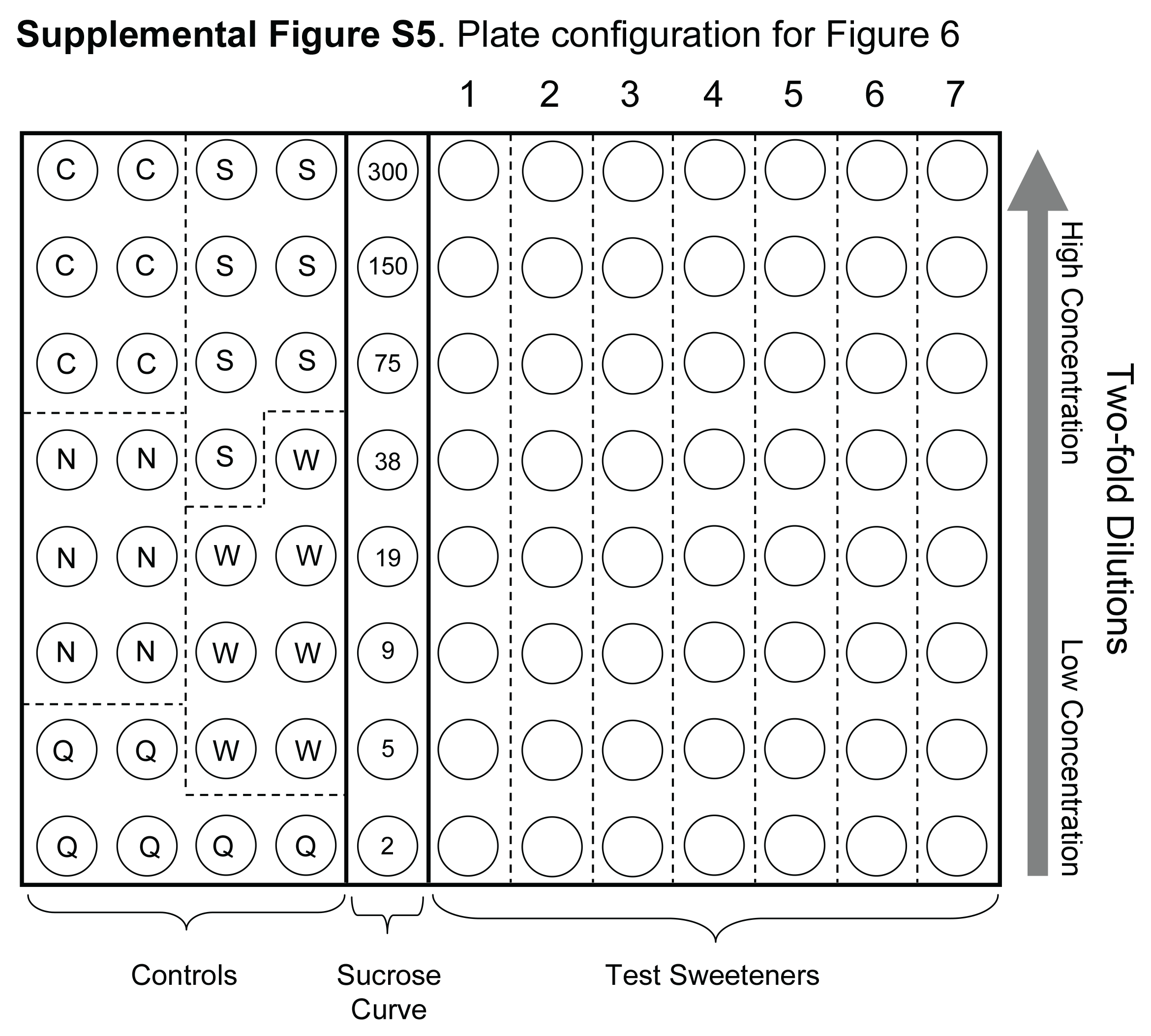

Supplement: Figure S5 — Plate configuration for Figure 6. The figure shows a schematic diagram of the 96-well plate, and the contents of each well, used for the experiment. S=100 mM sucrose, Q=1 mM quinine, N=100 mM NaCl, C = 10 mM citric acid, W = water. Numeric values are the concentrations in mM of sucrose. See Figure 6 for concentrations of test sweeteners. (TIF) [file pone.0072391.s007.tif]

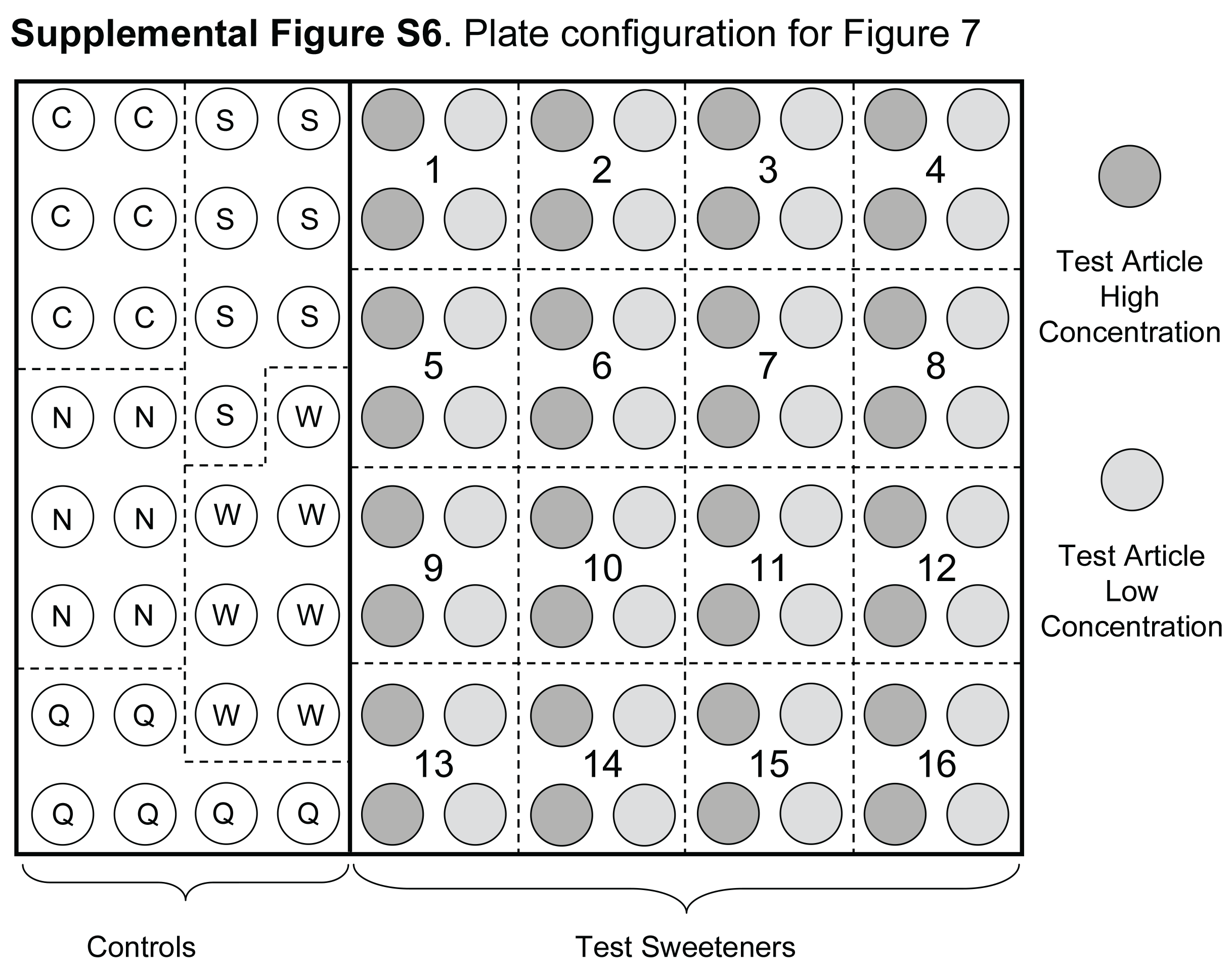

Supplement: Figure S6 — Plate configuration for Figure 7. The figure shows a schematic diagram of the 96-well plate, and the contents of each well, used for the experiment. S=100 mM sucrose, Q=1 mM quinine, N=100 mM NaCl, C = 10 mM citric acid, W = water. Numeric values are the concentrations in mM of sucrose. See caption to Figure 7 for concentrations of test articles. (TIF) [file pone.0072391.s008.tif]

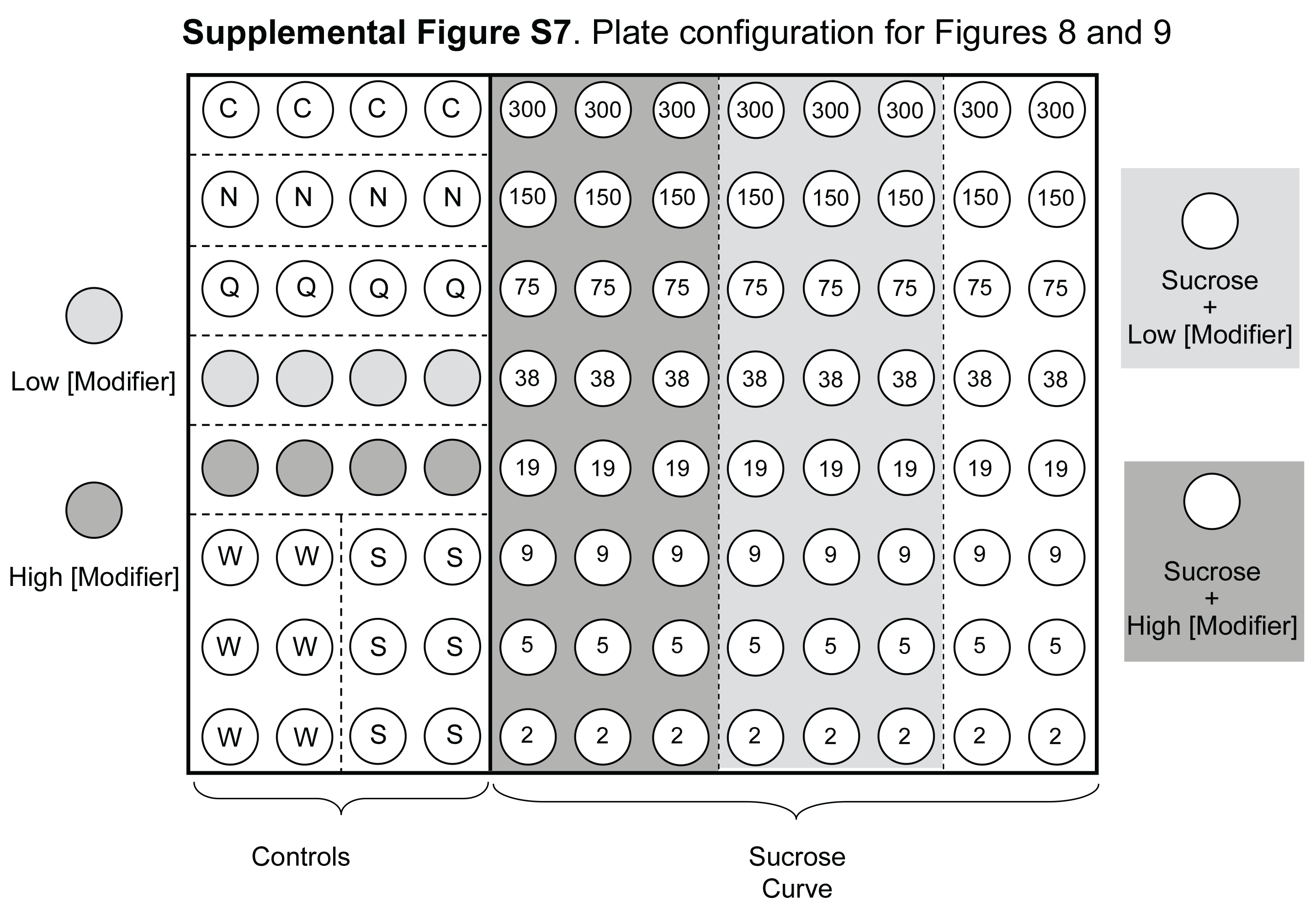

Supplement: Figure S7 — Plate configuration for Figures 8 and 9. The figure shows a schematic diagram of the 96-well plate, and the contents of each well, used for the experiment. S=100 mM sucrose, Q=1 mM quinine, N=100 mM NaCl, C = 10 mM citric acid, W = water. Numeric values are the concentrations in mM of sucrose. See Figures 8 and 9 for concentrations of modifiers (alloxan and ZnSO4, respectively). (TIF) [file pone.0072391.s009.tif]

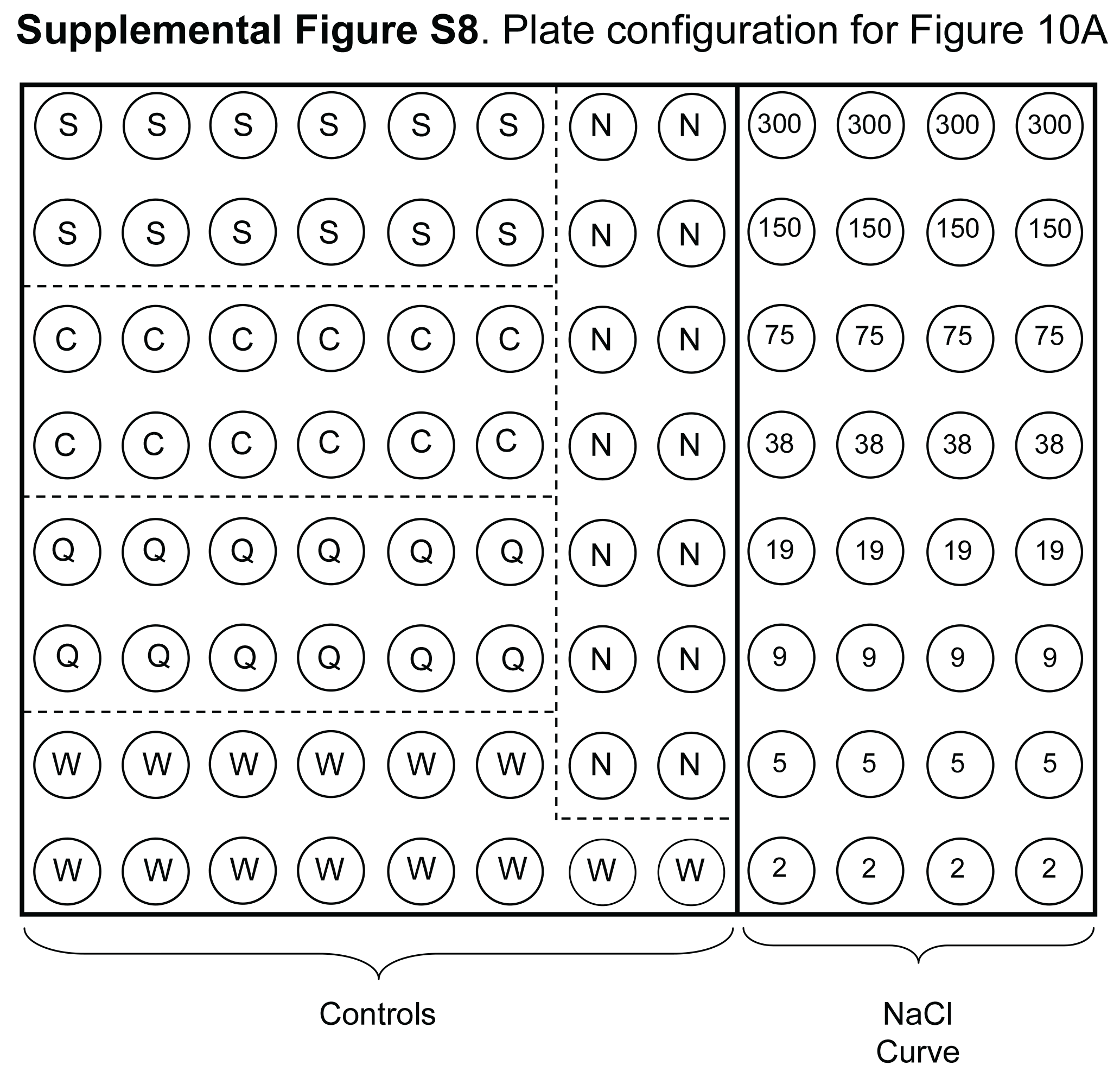

Supplement: Figure S8 — Plate configuration for Figure 10A. The figure shows a schematic diagram of the 96-well plate, and the contents of each well, used for the experiment. S=100 mM sucrose, Q=1 mM quinine, N=100 mM NaCl, C = 10 mM citric acid, W = water. Numeric values are the concentrations in mM of NaCl. (TIF) [file pone.0072391.s010.tif]

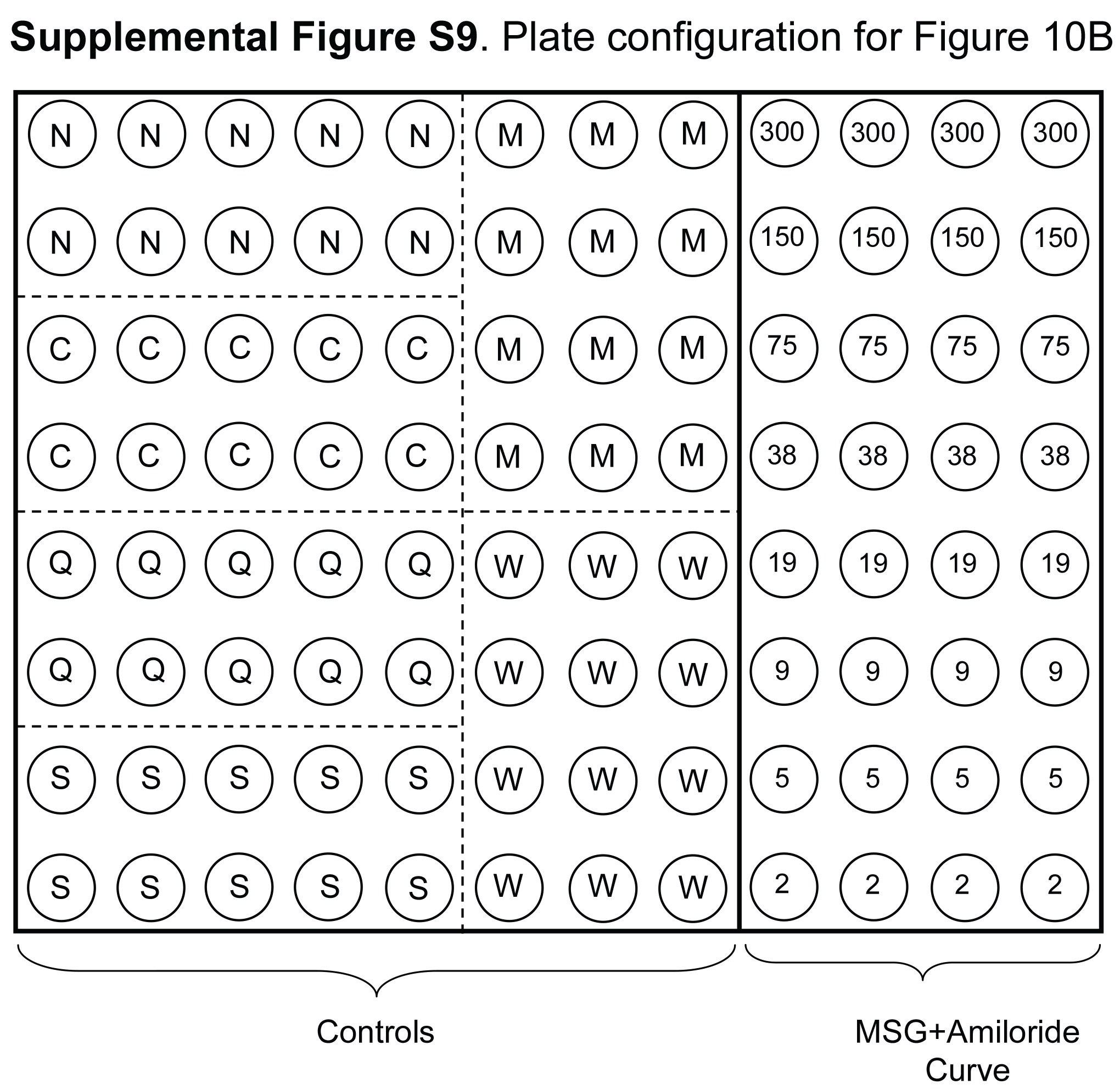

Supplement: Figure S9 — Plate configuration for Figure 10B. The figure shows a schematic diagram of the 96-well plate, and the contents of each well, used for the experiment. S=100 mM sucrose, Q=1 mM quinine, N=100 mM NaCl, C = 10 mM citric acid, W = water, M=100 mM MSG+100 µM amiloride. Numeric values are the concentrations in mM of MSG. 100 µM amiloride was added to each concentration of MSG in the concentration range. (TIF) [file pone.0072391.s011.tif]

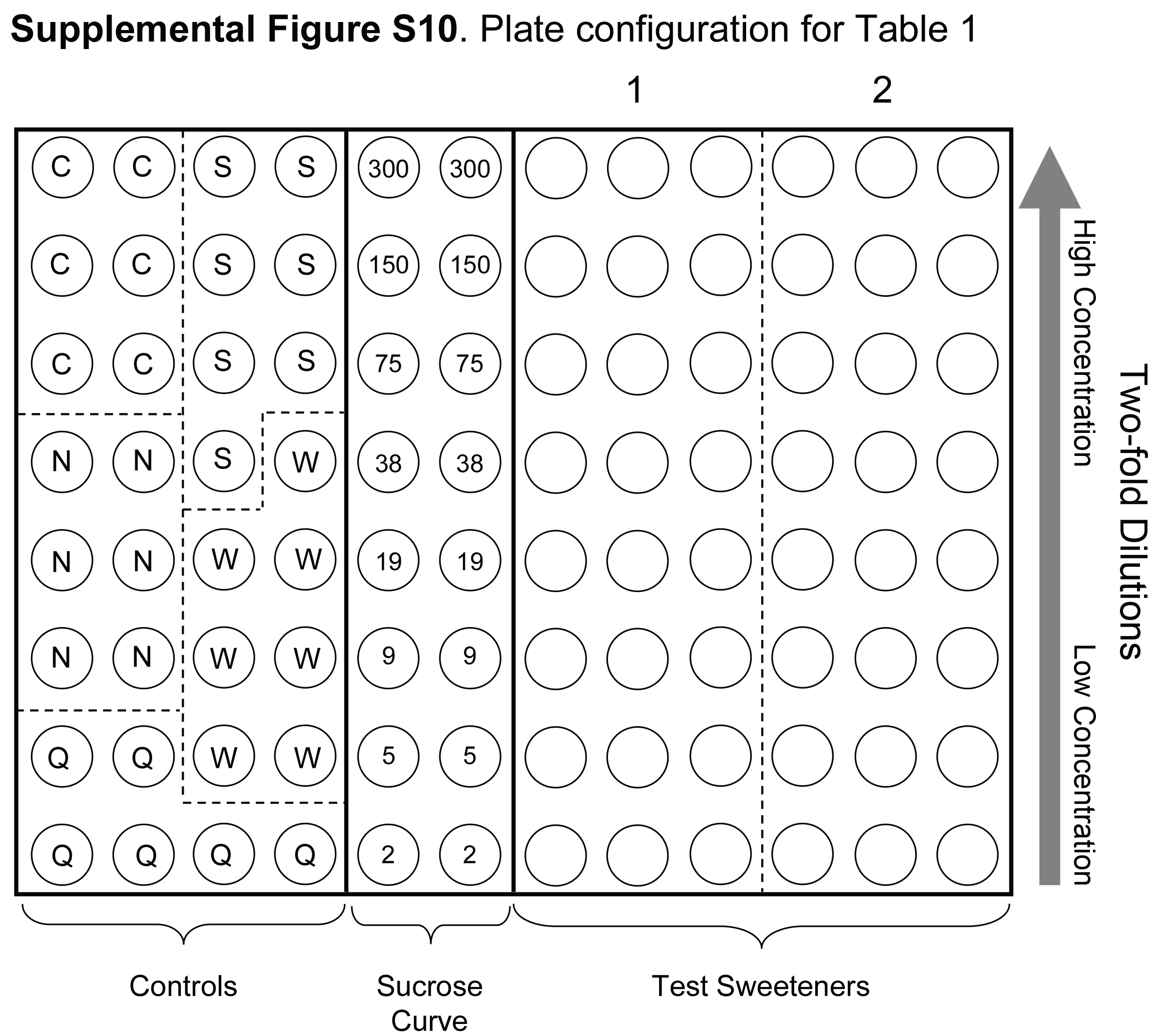

Supplement: Figure S10 — Plate configuration for Table 1. The figure shows the general template for the plate configuration used to generate data given in the table. S=100 mM sucrose, Q=1 mM quinine, N=100 mM NaCl, C = 10 mM citric acid, W = water. Numeric values are the concentrations in mM of sucrose. Concentration ranges for test sweeteners were obtained by successive 2-fold dilutions from the maximally effective concentrations of each. (TIF) [file pone.0072391.s012.tif]
